# *Pten* haploinsufficiency disrupts scaling across brain areas during development in mice

**DOI:** 10.1038/s41398-019-0656-6

**Published:** 2019-12-05

**Authors:** Amy E. Clipperton-Allen, Ori S. Cohen, Massimiliano Aceti, Aya Zucca, Jenna Levy, Jacob Ellegood, Jason P. Lerch, Damon T. Page

**Affiliations:** 1 Department of Neuroscience, The Scripps Research Institute, Jupiter, FL USA; 2 Mouse Imaging Centre, Hospital for Sick Children, Toronto, ON Canada; 3 Wellcome Centre for Integrative Neuroimaging, University of Oxford, Oxford, UK; 4 Present Address: Drug Discovery, Moffitt Cancer Center, Tampa, FL USA

**Keywords:** Autism spectrum disorders, Molecular neuroscience

## Abstract

Haploinsufficiency for *PTEN* is a cause of autism spectrum disorder and brain overgrowth; however, it is not known if *PTEN* mutations disrupt scaling across brain areas during development. To address this question, we used magnetic resonance imaging to analyze brains of male *Pten* haploinsufficient (*Pten*^+/−^) mice and wild-type littermates during early postnatal development and adulthood. Adult *Pten*^+/−^ mice display a consistent pattern of abnormal scaling across brain areas, with white matter (WM) areas being particularly affected. This regional and WM enlargement recapitulates structural abnormalities found in individuals with *PTEN* haploinsufficiency and autism. Early postnatal *Pten*^+/−^ mice do not display the same pattern, instead exhibiting greater variability across mice and brain regions than controls. This suggests that *Pten* haploinsufficiency may desynchronize growth across brain regions during early development before stabilizing by maturity. *Pten*^+/−^ cortical cultures display increased proliferation of glial cell populations, indicating a potential substrate of WM enlargement, and provide a platform for testing candidate therapeutics. *Pten* haploinsufficiency dysregulates coordinated growth across brain regions during development. This results in abnormally scaled brain areas and associated behavioral deficits, potentially explaining the relationship between *PTEN* mutations and neurodevelopmental disorders.

## Introduction

Autism spectrum disorder (ASD) is a common neurodevelopmental disorder (1:59 children in USA^1^) characterized by deficits in social behavior and communication, and restricted, repetitive behavior and interests^2^. As it is a polygenic disorder that presents with great symptomatic variability, biomarkers, such as macrocephaly (head circumference >2 SD above normal), may be useful to stratify the ASD population. Approximately 15–20% of the clinical ASD population show macrocephaly^3–5^. Of these, ~10–25%^6–12^ also have mutations in the gene *PTEN* (*Phosphatase and tensin homolog*), which is causative of macrocephaly/autism syndrome (MIM #605309).

Although *PTEN* mutations have been shown to induce macrocephaly and brain overgrowth, the pattern of enlargement at the level of individual brain areas during development has not been characterized. Individuals with *PTEN* mutations show brain enlargement and white matter (WM) abnormalities, some with changes in ventricular volume, vascular malformations, enlarged perivascular spaces, and/or gray matter (GM) heterotopia^13–17^. Individuals with ASD and *PTEN* mutations (*PTEN*-ASD) had considerably greater brain enlargement than those with ASD without a *PTEN* mutation (with or without macrocephaly) and healthy controls^14^. Additionally, the increased WM volume, along with increased WM hypointensities (suggestive of increased myelination), in *PTEN*-ASD were related to observed deficits in IQ, processing speed, and working memory^14^.

These data suggest that effects of *PTEN* mutations are not equivalently expressed throughout the brain. However, to date there have been no longitudinal or early-stage studies of the neuroanatomical developmental trajectories of these mutations. Due to technical issues of pre- and neo-natal magnetic resonance imaging (MRI), and because ASD is rarely diagnosed before 2 years of age, animal models are essential to address these questions. Germline haploinsufficient (*Pten*^+/−^) mice approximate the heterozygous missense or partial/complete loss-of-function mutations leading to *PTEN* haploinsufficiency in humans^8,14,18,19^. In addition to recapitulating a number of phenotypes, including social behavior deficits and increased repetitive behavior^20–22^, *Pten*^+/−^ mice show macroscale brain overgrowth from birth^20,22,23^.

In the current study, we used MRI in this mouse model of macrocephaly/autism syndrome at two developmental stages to explore pathological neuroanatomical trajectories that affect scaling of individual brain regions. We analyzed regional brain volume at postnatal day 7 (P7), which approximates human birth^24,25^, and is the age with the lowest postnatal difference in brain mass between *Pten*^+/−^ mice and wild-type littermate controls (*Pten*^+/+^)^23^. We also examined brain region volume in mature animals (P60 adults). These data provide an opportunity to associate cellular and behavioral phenotypes with these neuroanatomical phenotypes in *Pten*^+/−^ mice.

## Materials and methods

### Subjects

Mice of the *B6.129-Pten*^*tm1Rps*^ line were used, as previously described^20,26^ (see Supplementary Methods). All research was approved by The Scripps Research Institute’s Institutional Animal Care and Use Committee and conducted in accordance with National Institutes of Health and Association for Assessment and Accreditation of Laboratory Animal Care International (AAALAC) guidelines.

### Magnetic resonance imaging

MRI was performed on perfused brains of male P7 [*Pten*^+/+^ (*n* = 10) and *Pten*^+/−^ (*n* = 10) from 7 litters] and P60 [*Pten*^+/+^ (*n* = 10) and *Pten*^+/−^ (*n* = 9) from 6 litters] mice as previously described^27^ (see Supplementary Methods). All analyses were performed blind to genotype.

### Eriochrome cyanine R. staining for myelin

Consecutive coronal sections (60 μm thick) from fixed brains of adult male *Pten*^+/+^ (*n* = 3) and *Pten*^+/−^ (*n* = 4) littermates were stained with eriochrome cyanine R. to visualize myelin (see Supplemental Methods). Acquired images were quantified (blind for genotype) for corpus callosum (CC) width, anterior commissure (AC) width, and fornix area through anterior/posterior axis positions (estimated with The Mouse Brain in Stereotaxic Coordinates, 3^rd^ edition^28^) using Olympus VS-DESKTOP software (Olympus, Centerville, PA).

### Isotropic fractionator and flow cytometry

We used isotropic fractionation and flow cytometry on fixed P7 *Pten*^+/+^ (*n* = 3) and *Pten*^+/−^ (*n* = 3) brains as has been previously described to calculate the number and percent of total nuclei positive or negative for neuronal nuclei marker NeuN or oligodendrocyte lineage marker oligodendrocyte transcription factor 2 (Olig2)^29^ (see Supplementary Methods). All analyses were performed blind to genotype.

### Cell culture and immunocytochemistry primary cortical culture

Cortices of P0 mice (*n* = 3 per genotype) were dissected and individually plated on coverslips pre-coated with poly-D-lysine (Thermo Fisher Scientific, Asheville, NC) in 12-well plates (500,000 cells per well). Cells were grown in a 37 °C incubator with 5% CO_2_ levels. Each cortex was kept separate throughout the experiment in order to obtain true biological replicates. All P0 animals were from the same litter and each cortex was plated on multiple coverslips. See Supplementary Methods for details.

#### PTEN-Long

The JpExress404 *PTEN-Long* construct (containing a V5/His tag), deposited by Ramon Parsons^30^, was purchased from Addgene (Cambridge, MA). Protein was resolved by SDS-PAGE electrophoresis and quantified by comparison to bovine serum albumin (BSA) standard (Pierce) using Imperial Protein Stain (Thermo Fisher Scientific). Additional details in Supplementary Methods.

#### Culture treatment and immunocytochemistry

To measure cell cycle re-entry and gliogenesis, we performed 24 h 5-bromo-2′deoxyuridine (BrdU) pulse-chase assays at 7 or 11 days-in-vitro (DIV7 or DIV11), co-applying vehicle (0.1% ethanol; Thermo Fisher Scientific), 10 μM phosphoinositide-3-kinase (PI3K) inhibitor LY294002 (S1105, Selleck Chemicals, Houston, TX), or 10 nM PTEN-Long. After 24 h treatment, cultures were fixed and stained with primary (anti-BrdU, anti-Ki67, anti-Olig2, anti-Sox9, and anti-NeuN) and secondary antibodies, with DAPI (1:12500) to identify nuclei (antibody details in Supplementary Methods). For each staining combination, three randomly selected 180,000–200,000 µm^2^ areas from each coverslip (one mouse per coverslip) were analyzed and averaged per animal. All cell counts were performed blind to genotype and treatment.

### Statistical analysis

MRI data were analyzed using False Discovery Rate (FDR)-corrected independent-sample *t*-tests^31^ to compare genotypes; *p* values are listed in Supplementary Table S1. Specific values of all other statistical tests, including effect sizes, are in Supplementary Table S2. Thus, neither are included in the text. See Supplementary Methods for details of statistical analyses.

Power analyses (G*Power^32^) and previous experience were used to determine sample size for each assay. The α level was set at 0.05, all tests were two-tailed, and all statistics were performed after passing normality tests using PASW 18 (IBM Corporation, Armonk, NY).

## Results

### *Pten* haploinsufficiency induces large absolute volume changes across brain areas in early postnatal and adult mice

**Fig. 1:**
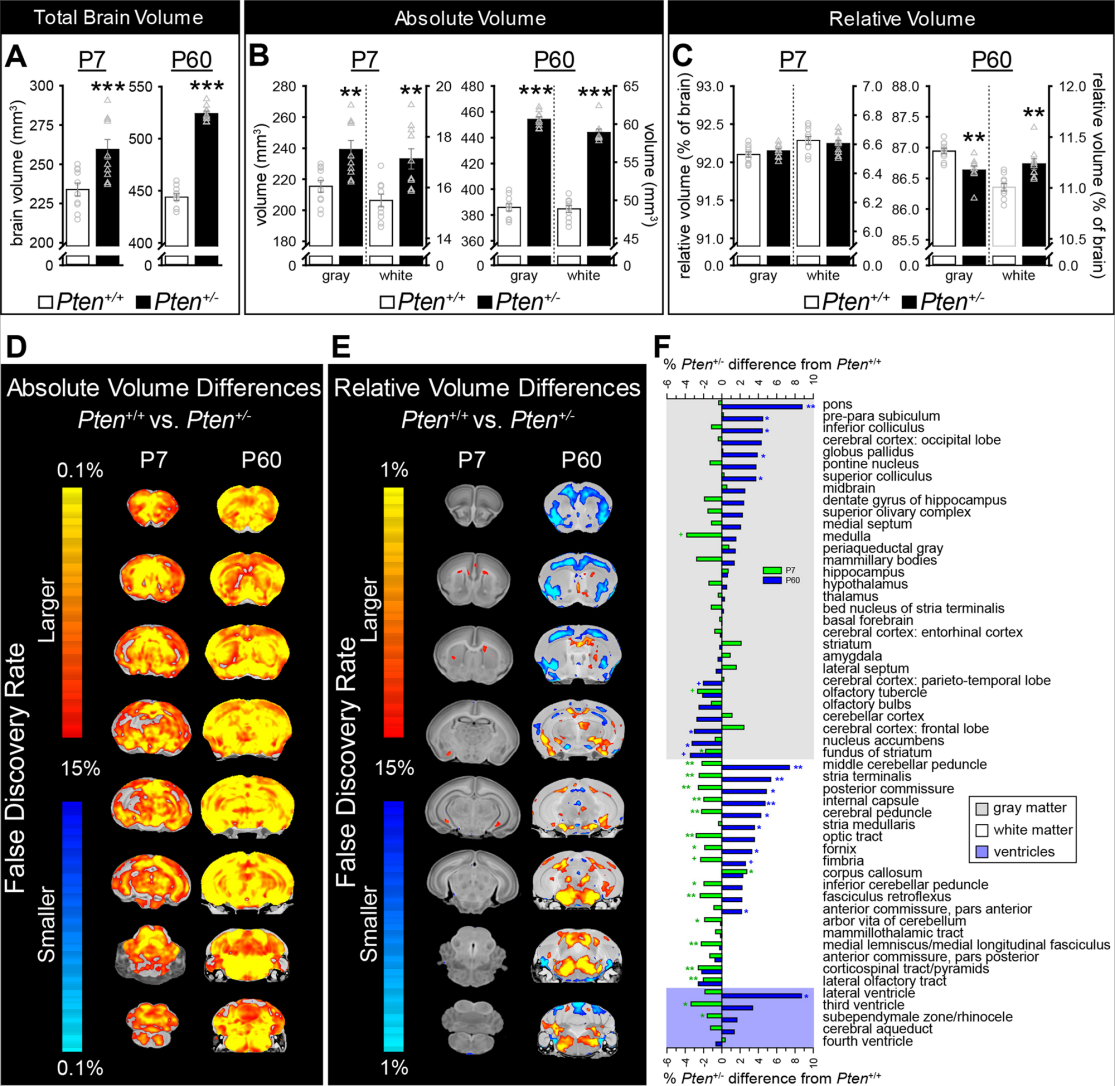
Absolute brain volume is larger in *Pten*^+/−^ mice (black bars; P7: *n* = 10; P60: *n* = 9) than *Pten*^+/+^ controls (white bars; both ages: *n* = 10) at postnatal day 7 (P7) and P60, but relative volume changes differ by brain region, including relative enlargement of white matter (WM) and relative reduction in volume of gray matter (GM) at P60. **a** Total brain volume at P7 and P60. **b** Absolute volume of GM and WM at P7 and P60. **c** Relative volume of GM and WM at P7 and P60. **d**, **e** Magnetic resonance imaging fly-through of absolute (**d**) and relative (**e**) volume differences in *Pten*^+/−^ mice at P7 and P60. **f** Relative volume difference between *Pten*^+/+^ and *Pten*^+/−^ mice [(*Pten*^+/−^ volume) – (*Pten*^+/+^ volume)/(*Pten*^+/+^ volume) × 100] at P7 (green) and P60 (blue) in individual GM (gray backfill), WM (white backfill), and ventricular (pale violet backfill) brain regions. Green symbols indicate differences at P7, blue symbols indicate differences at P60. ***p < 0.001, **p < 0.01, *p < 0.05, +*p* < 0.1. Mean ± SEM.

**Fig. 2:**
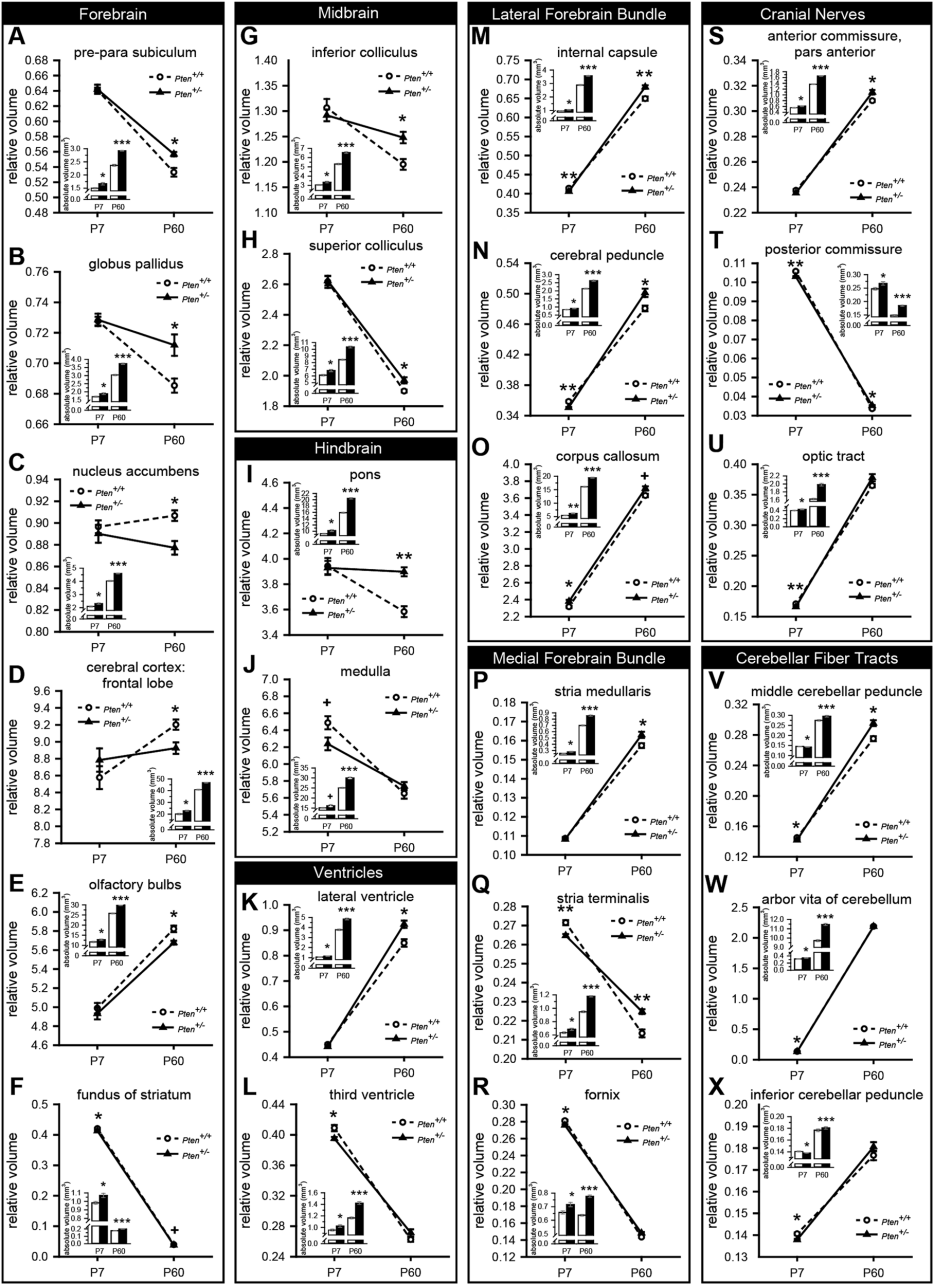
Absolute and relative volumetric changes at postnatal day 7 (P7; *Pten*^+/+^*n* = 10; *Pten*^+/-^*n* = 10) and P60 (*Pten*^+/+^*n* = 10; *Pten*^+/-^*n* = 9) in *Pten*^+/+^ (white bars/circles) and *Pten*^+/−^ (black bars/triangles) mice for brain regions with the largest relative changes in volume. **a–j** Gray matter regions in the forebrain (**a–f**), midbrain (**g**, **h**), and hindbrain (**i**, **j**). **a–f** Forebrain regions showed both increases (pre-para subiculum, **a**; globus pallidus, **b** and decreases (nucleus accumbens, **c**; cerebral cortex: frontal lobe, **d**; olfactory bulbs, **e**) in relative volume at P60, with the fundus of striatum (**f**) being the only brain region in which both P7 and P60 *Pten*^+/−^ mice have relative decreases in volume (approaching but not reaching significance at P60). **g**, **h** Midbrain (inferior colliculus, **g**; superior colliculus, **h)** regions only showed relative increases in adulthood, and no changes at P7. **i**, **j** The largest structure in the hindbrain (pons, **i**) was relatively increased in adulthood, and the medulla (**j**) showed a trend to a relative volume decrease at P7. **k**, **l** The lateral ventricle was relatively increased at P60 (**k**), and the third ventricle was relatively decreased at P7 (**l**). **m–o** All relatively changed white matter regions in the lateral forebrain bundle (LFB) were relatively increased at P60. At P7, there were decreases in LFB regions (internal capsule, **m**; cerebral peduncle, **n**), as well as an increase in the corpus callosum (**o**; the only brain region with a relative increase in volume in both P7 and P60 *Pten*^+/−^ mice, although it only reaches the level of a trend at P60). All P7 white matter regions showing differences in the medial forebrain bundle (stria medullaris, **p**; stria terminalis, **q**; fornix, **r**), cranial nerves (anterior commissure, pars anterior, **s**; posterior commissure, **t**; optic tract, **u**), and cerebellar fiber tracts (middle cerebellar peduncle, **v**; arbor vita of cerebellum, **w**; inferior cerebellar peduncle, **x**) were relatively decreased. ***p < 0.001, **p < 0.01, *p < 0.05, +*p* < 0.1. Mean ± SEM.

Total brain volume in *Pten*^+/−^ mice, measured by MRI, was 10.9% larger than *Pten*^+/+^ controls at P7 and 18.1% larger at P60, as expected^20,22,23^ (Fig. 1a). Absolute volume increases in GM (P7: 11.0%; P60: 17.7%; Fig. 1b, d), WM (P7: 10.6%; P60: 20.6%; Fig. 1b, d), and almost all measured structures were found in *Pten*^+/−^ mice at both ages (see Figs. 1, 2; Supplementary Figs. S1 and S2, Table S1). An overview of absolute volume changes is shown in Fig. 1d. There were no litter effects on brain volume at either age (Supplementary Fig. S3A, B). There were no genotype differences in body mass, although it was significantly affected by litter at P7 (with a trend to significance at P60; Supplementary Fig. S3C, D).

These results indicate that gross brain volume is increased at both P7 and P60 in *Pten*^+/−^ mice. These increases are remarkably similar to those in brain mass (P7: ~8%; P60: ~20%)^23^. Detailed segmentation showed that GM, WM, and virtually all individual brain regions were also increased in *Pten*^+/−^ mice at both timepoints.

### Dynamic relative volume changes during postnatal development in the *Pten* haploinsufficient brain

While brains of *Pten*^+/−^ mice are clearly enlarged at both ages, absolute volume analysis did not determine if they were larger but normally scaled, or if individual regions were more or less overgrown (abnormally scaled) relative to whole brain volume change. This is an important question, as enlarged but normally scaled brains may not be pathological (not all macrocephalic or megalencephalic individuals have neurodevelopmental disorders). Abnormal scaling could indicate abnormal connectivity, ectopic tissue, or excess or inadequate enervation of important brain areas, any of which could contribute to behavioral phenotypes. Additionally, abnormally scaled regions could indicate developmental timing of the effects of *Pten* mutations. To assess scaling of individual brain regions, we calculated the relative volume of each brain region ([(brain region volume)/(whole brain volume) × 100]; see Fig. 1e for overview). While this is a commonly used measure, it requires some caveats. First, different brain areas do not necessarily scale on a one-to-one ratio (e.g., cerebellum scales with brain volume in a non-linear manner^33^). Additionally, large scale differences in one brain area may artificially increase or decrease differences in other regions when normalized by total brain volume (e.g., a 30% decrease in cerebellum size may cause an artificial increase in forebrain volume). Thus, one must also consider absolute volume differences when comparing genotypes.

Combining individual regions into GM and WM categories revealed a developmental change: there were no relative differences in either category at P7 (Fig. 1c, e), but at P60 WM was relatively increased and GM was relatively decreased in *Pten*^+/−^ mice (Fig. 1c, e). To explore this developmental change, and look for patterns of abnormal scaling, we performed a more in-depth analysis of the relative volume of individual GM, WM, and ventricular brain regions.

### Gray matter regions

In P60 *Pten*^+/−^ mice, 32% (11/34) of GM regions analyzed showed abnormal scaling (Supplementary Fig. S4C). Only forebrain regions showed relative decreases (Fig. 2c–f; Supplementary Fig. S1A, S4A), while the relatively increased regions were distributed across all three GM divisions (Fig. 2a, b, g–i; Supplementary Fig. S4A, C), with the largest relative increase in the pons (hindbrain; Fig. 2i), which showed the largest percent change in relative volume of any individual brain structure (Fig. 1f; Supplementary Table S1).

In P7 *Pten*^+/−^ mice, however, only 12.5% (4/32) of GM regions were relatively decreased, with no relative increases (Supplementary Fig. S4B, C). These changes were restricted to the forebrain and hindbrain, with a lower relative decrease (2.4%) over three regions in the forebrain (Fig. 2f; Supplementary Fig. S1M, Table S1) than the medulla (3.8%), the sole decreased hindbrain region (Fig. 2j; Supplementary Fig. S4B).

### White matter regions

A larger proportion of WM than GM regions showed abnormal scaling in *Pten*^+/−^ mice of both ages. At P60, relative volume increases were seen in 48% (11/23) of WM regions (Supplementary Fig. S4C), with no relative decreases found (Fig. 2m–q, s, t, v; Supplementary Fig. S4A, C, Table S1). These WM regions were distributed across lateral (LFB; Fig. 2m–o) and medial (MFB; Fig. 2p, q) forebrain bundles, cranial nerves (Fig. 2s, t; Supplementary Table S1), and cerebellar fiber tracts (Fig. 2v; Supplementary Table S1).

An even greater proportion (79%; 15/19) of WM regions showed relative volume changes at P7 (Supplementary Fig. S4C), all but one in the opposite direction to those at P60 (Supplementary Fig. S4B, C). The CC (Fig. 2o), in the LFB, was the only region to show a relative increase, but the size of the region (more than twice the volume of any other P7 WM region) and the magnitude of its change (2.7%, one of the largest relative volume changes in WM regions at P7) were sufficient to counteract the impact of all 14 relatively decreased WM regions (Fig. 2m, n, q, r, t–x; Supplementary Fig. S2I–K, N, O, S4B), resulting in no relative P7 WM differences overall.

### Ventricles

The ventricles also showed some abnormal scaling: at P60, lateral ventricles were 8.7% increased (Fig. 2k), and the third ventricle and subependymale zone/rhinocele were relatively decreased at P7 (Fig. 2l; Supplementary Fig. S2F, S4C).

While absolute volume is increased in *Pten*^+/−^ mice across brain regions at both P7 and P60, neither GM nor WM show relative differences at P7, while at P60 GM is relatively undergrown but WM is relatively overgrown. P7 mice show very few GM changes, but the many WM area decreases are counteracted by the increased CC, resulting in no overall WM or GM changes. In P60 mice, however, there are no relative decreases in WM, and the GM decrease is driven by the forebrain.

### *Pten* haploinsufficiency leads to increased variability across mice and brain regions in early postnatal animals

**Fig. 3:**
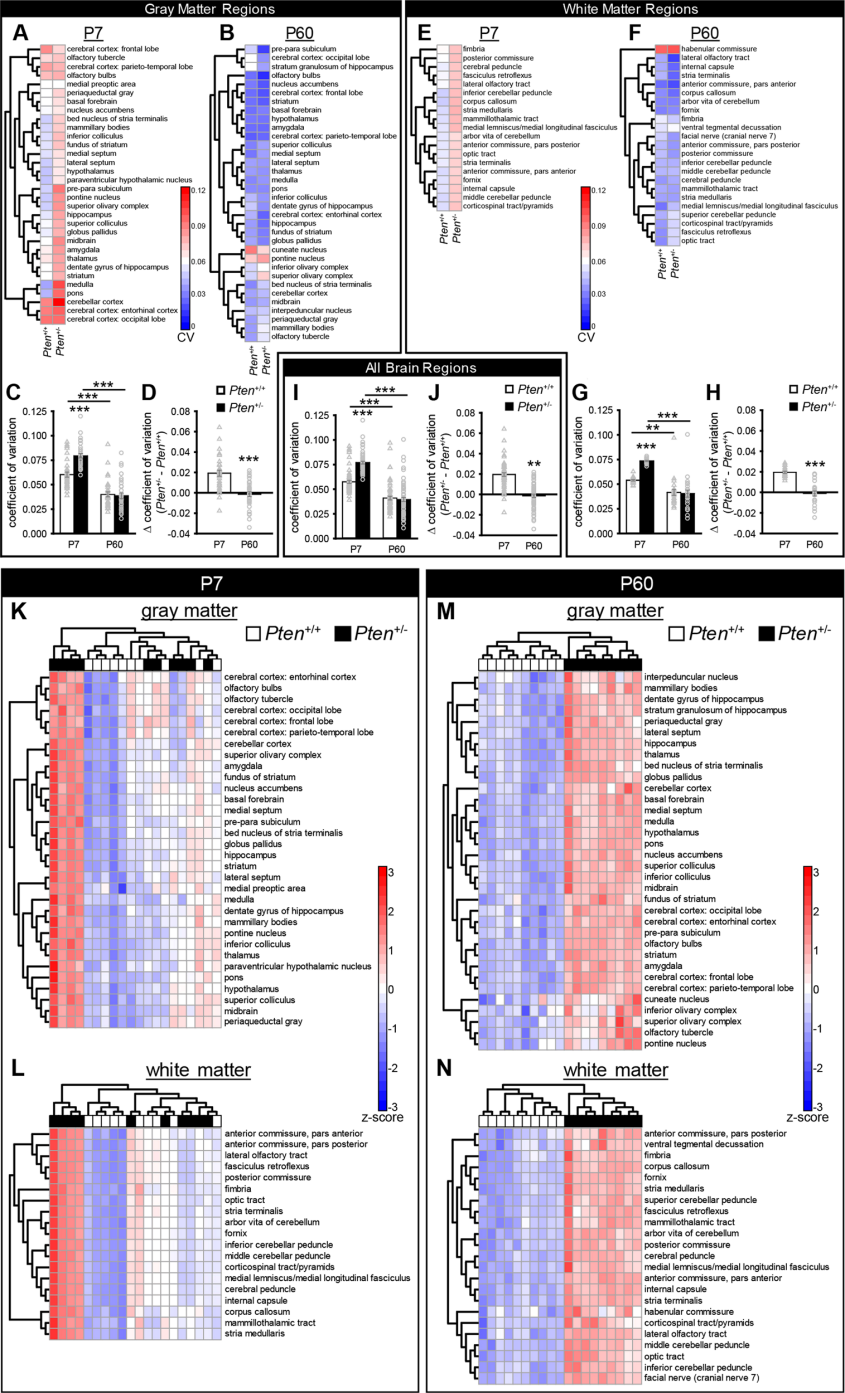
*Pten*^+/-^ mice show higher variation than *Pten*^+/+^ mice at postnatal day 7 (P7; *Pten*^+/+^*n* = 10; *Pten*^+/−^*n* = 10) but not P60 (*Pten*^+/+^*n* = 10; *Pten*^+/−^*n* = 9), both genotypes show a decrease in variation from P7 to P60, and unbiased clustering of deviation from age group mean shows clear segregation of genotypes at postnatal day 60 but not at P7. **a**, **b** Heatmaps of the coefficient of variation [CV = (standard deviation_age+genotype group_)/(mean_age+genotype group_)] for each gray matter brain region across each genotype at P7 (**a**) and P60 (**b**). **c** Average CV across gray matter regions at P7 and P60. **d** Difference between *Pten*^+/−^ (black bars) and *Pten*^+/+^ (white bars) CVs for gray matter regions at P7 and P60. **e**, **f** Heatmaps of the CV for each white matter region across each genotype at P7 (**e**) and P60 (**f**). **g** Average CV across white matter regions at P7 and P60. **h** Difference between *Pten*^+/−^ and *Pten*^+/+^ CVs for white matter regions at P7 and P60. **i** Average CV across all brain regions is higher in *Pten*^+/−^ mice at P7 than *Pten*^+/−^ mice at P60, and both *Pten*^+/+^ and *Pten*^+/−^ mice show lower CVs at P60 than P7. **j** The difference between genotypes in average CV across all brain regions is lower at P60 than at P7. **k–n** Heatmaps of the deviation from age group mean, calculated as *z*-score [z-score = (volume_mouse_ – mean volume_all P7 mice_)/(standard deviation_all P7 mice_)]. **k**, **l** No clustering by genotype in P7 mice for gray matter (**k**) or white matter (**l**) brain regions. **m**, **n** P60 mice do cluster by genotype for for gray matter (**m**) and white matter (**n**) brain regions. Left dendrograms indicate unbiased clustering of brain regions. Top dendrogram indicates unbiased clustering of individual mice (white, *Pten*^+/+^; black, *Pten*^+/−^). ***p < 0.001, **p < 0.01. Mean ± SEM.

Structural analyses of individuals with ASD, and those with macrocephaly/autism syndrome, have suggested they might show greater variation than neurotypical individuals (e.g., Frazier et al.^14^,). To ascertain if this increased variability was present in our mouse model, we calculated the coefficient of variation (CV) among mice in each age and genotype category for each brain region [e.g., CV = (SD_P7 *Pten*+/−_)/(mean_P7 *Pten*+/−_)]. This produced heatmaps (Fig. 3a, b, e, f) showing the degree of variation across mice of a given age and genotype (e.g., P7 *Pten*^+/−^). To determine if there were different degrees of overall variability between groups, we averaged the CVs of the brain regions to produce an average variability measure for each age and genotype group across WM regions (Fig. 3g, h), GM regions (Fig. 3c, d), and all regions together (Fig. 3i, j). We then compared the average group CVs and found that while both *Pten*^+/+^ and *Pten*^+/−^ mice showed reductions in variation from P7 to P60, there were genotype differences only at P7 (Fig. 3a, c, e, g, i). Consistently, the difference in variation between *Pten*^+/+^ and *Pten*^+/−^ mice (ΔCV = CV_*Pten+/*−_ − CV_*Pten+/+*_) decreased from P7 to P60 (Fig. 3d, h, j).

As increased variability was observed at P7, particularly in *Pten*^+/−^ mice, and the genotype difference in absolute brain volume is lowest at this age, we hypothesized that differences between animals of different genotypes would be less clear at P7 than P60. Thus, we used unbiased clustering to determine which regions and mice were most related to each other, based on heatmaps of *z*-score from either an age or a genotype group mean (as a variability index). As proof of concept, we first clustered *Pten*^+/+^ mice or *Pten*^+/−^ mice by age (Supplementary Fig. S5). Since mouse brains are markedly smaller at P7 than P60 in both genotypes (Fig. 1a), all P7 brains should show negative *z*-scores, and all P60 brains should have positive *z*-scores, clearly segregating the ages. This is what we observed (Supplementary Fig. S5), confirming that unbiased clustering analysis of *z*-scores can successfully classify distinct groups of mice. Using this analysis on each age group (Fig. 3k–n), we found that at P60 mouse clustering and genotype were unambiguously matched for both GM and WM (Fig. 3m, n). However, subject clustering within P7 mice did not reflect genotypic identity for either GM or WM (Fig. 3k, l). While this could be due to a lack of mean differences, all brain regions analyzed were significantly enlarged in *Pten*^+/−^ mice at P7 (with the exception of the medulla, which showed a trend to enlargement). At neither age did mice cluster by litter.

Taken together, these results suggest that there is more variability in volume changes across mice and brain areas at P7 than P60, and that this is further increased in P7 *Pten*^+/−^ mice. This resulted in more clear-cut distinctions between genotypes at P60. The increased variability between animals at P7, and the inability to cluster mice of this age by genotype, is consistent with the decreased difference in overall brain volume and lack of differences in relative GM and WM volumes at P7.

### Histological confirmation of white matter abnormalities in the *Pten* haploinsufficient brain

**Fig. 4:**
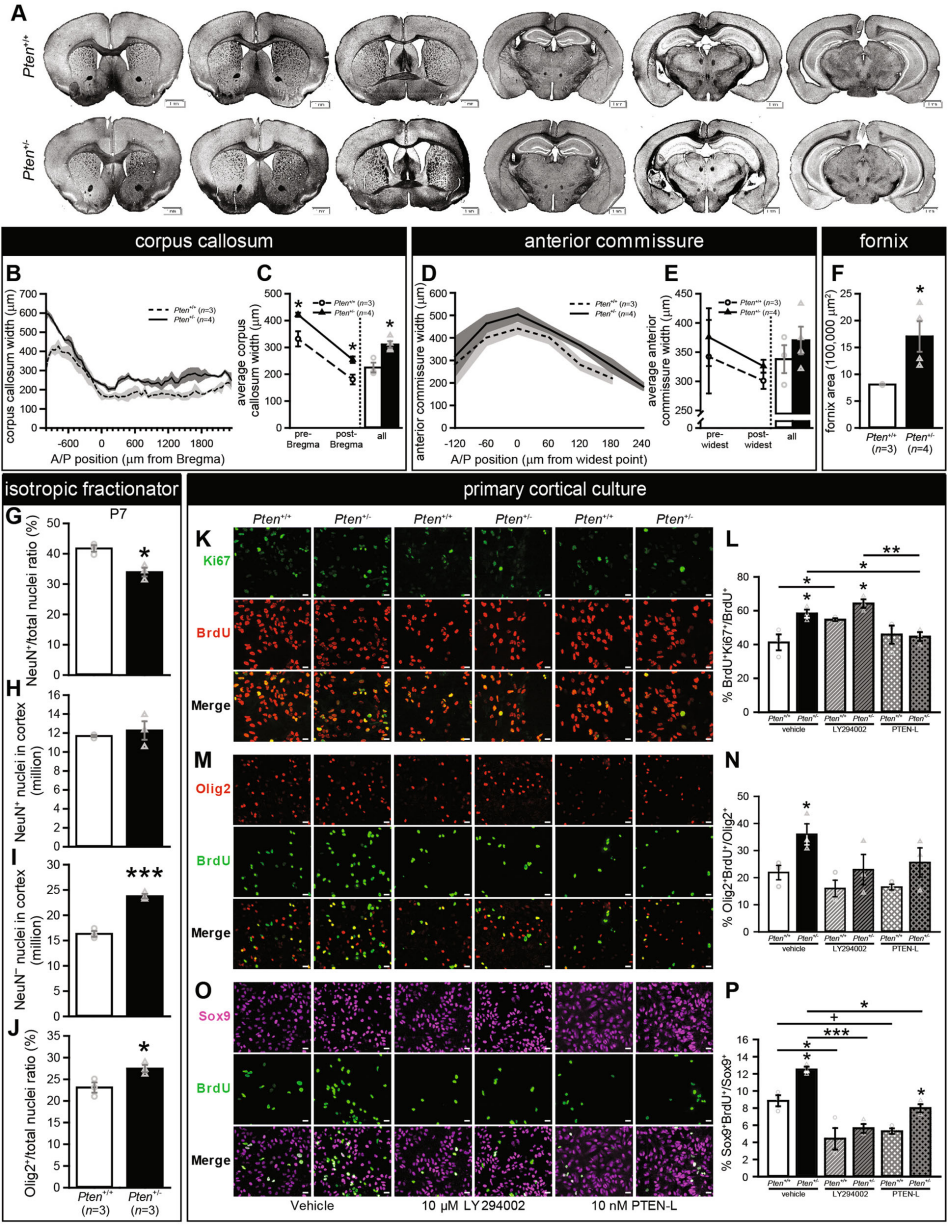
White matter tracts are larger in adult *Pten*^+/−^ mice, and *Pten*^+/−^ mice show excess glia in vivo at postnatal day 7 (P7) and increased glial proliferation in culture. **a** Representative eriochrome cyanine R.-stained coronal sections from *Pten*^+/+^ (top) and *Pten*^+/−^ (bottom) adults. **b**, **c** *Pten*^+/−^ mice (black bars; *n* = 4) have wider corpus callosa than *Pten*^+/+^ mice (white bars; *n* = 3), measured at the midline. **d**, **e** The anterior commissure was not significantly wider in *Pten*^+/−^ mice when measured at the midline. **f** *Pten*^+/−^ mice have an enlarged fornix area. *p < 0.05. Mean ± SEM (gray shading and error bars). Scale bars, 1 mm. **g**–**j**) Isotropic fractionator and flow cytometry results in P7 *Pten*^+/+^ (white bars) and *Pten*^+/−^ (black bars) mice. **g** Percentage of nuclei positive for neuronal marker NeuN. **h** Number of NeuN^+^ nuclei. **i** Number of NeuN^–^ nuclei. **j** Percentage of nuclei positive for oligodendrocyte lineage marker Olig2. **k**–**p** Results of 5-bromo-2′deoxyuridine (BrdU) pulse-chase in primary cortical culture at 8 days-in-vitro (DIV8; **k**, **l**) or DIV12 (**m**–**p**). **k**, **l** Representative images (**k**) and quantification (**l**) of Ki67 and BrdU immunocytochemistry in cultures treated with vehicle, 10 μM PI3K inhibitor LY294002 (diagonal line pattern), or 10 nM PTEN-Long (crosshatched line pattern). **m, n** Representative images (**m**) and quantification (**n**) of Olig2 and BrdU immunocytochemistry in cultures treated with vehicle, 10 μM LY294002, or 10 nM PTEN-Long. **o**, **p** Representative images (**o**) and quantification (**p**) of immunocytochemistry for BrdU and astrocyte marker Sox9 in cultures treated with vehicle, 10 μM LY294002, or 10 nM PTEN-Long. All *n* = 3 biological replicates. ***p < 0.001, **p < 0.01, *p < 0.05, + *p* < 0.1. Mean ± SEM. Scale bars, 50 μm.

As MRI analysis indicated that relative enlargement was present in WM in adulthood, we used eriochrome cyanine R. staining to identify a selection of WM regions [CC, AC, and fornix] in adult *Pten*^+/+^ and *Pten*^+/−^ mice (Fig. 4a). Measurements of these areas revealed that CC width was increased in *Pten*^+/−^ mice overall and in sections both anterior and posterior to Bregma, and was also wider in anterior than posterior sections in both genotypes (Fig. 4b, c). There were no significant differences in average width of AC overall, or anterior or posterior to the widest point (Fig. 4d, e). The fornix showed a significant increase in area (Fig. 4f). These results demonstrate that enlargement of WM tracts identified via MRI can be detected using histological techniques.

### Increased gliogenesis as a potential substrate of *Pten* haploinsufficiency-induced white matter enlargement

Given that relative enlargement of WM was significant at P60 but not P7, we used isotropic fractionation in combination with flow cytometry to assess relative contributions of cells positive (putative neurons) and negative (putative glia) for NeuN in P7 cortices. In *Pten*^+/−^ mice, hyperplasia is the primary driver of increased brain mass, and we have previously shown that this is due to excess neurons at P0, but excess glia in adulthood^23^. However, it is unclear at which developmental timepoint this transition from neuronal to glial hyperplasia occurs. At P7, we found expected increases in overall brain mass, cortex mass, and total cortical nuclei (Supplementary Fig. S6A–C), with no effect on density (Supplementary Fig. S6D). Analysis of NeuN^+^ and NeuN^–^ nuclei revealed a significant decrease in NeuN^+^ percentage (Fig. 4g). This was due to increased NeuN^–^ nuclei number (Fig. 4i); no difference in NeuN^+^ nuclei was found (Fig. 4h). These results are consistent with the pattern of hyperplasia observed in adult, but not P0, *Pten*^+/−^ mice^23^. These data narrow the window for the change from excess neurons to excess glia to the first postnatal week. The increase in glial cell number likely contributes to the absolute WM enlargement seen in *Pten*^+/−^ mice at P7 (Fig. 1b).

To assess whether glial overgrowth was due to increased proliferation, and to test potential treatments, we performed primary culture of cells from cortices of P0 *Pten*^+/+^ and *Pten*^+/−^ mice. We used three mice per genotype and kept cultures from each mouse separate for true biological replicates.

### Primary cortical cultures derived from *Pten* haploinsufficient mice display increased markers of gliogenesis

We first confirmed that density of nuclei positive for Olig2, which is expressed in oligodendrocytes and their precursors^34,35^, was increased in *Pten*^+/−^ cultures at DIV12 (Supplementary Fig. S7A, B), showing concordance with the in vivo isotropic fractionator finding of increased percentage (Fig. 4j) and number (Supplementary Fig. S6E) of Olig2^+^ cells. We also found increased density of cells positive for Sex determining region Y-box 9 (Sox9; a transcription factor that is a nuclear marker for forebrain astrocytes outside neurogenic regions^36,37^) in these cultures at DIV12 (Supplementary Fig. S7D, E), and an increase in the percent of DAPI^+^ cells expressing Sox9 (Supplementary Fig. S7D, F). Interestingly, the percent of DAPI^+^ cells expressing Olig2 was decreased in *Pten*^+/−^ cultures, indicating that other cell types (e.g., Sox9^+^) are proliferating faster than Olig2^+^ cells (Supplementary Fig. S7A, C). We also verified that cells proliferating in these cultures, which were under glia-promoting conditions, were not neurons. Measuring NeuN^+^ cell density and percentage at DIV2 and DIV6 revealed that although total cell density increased with time (Supplementary Fig. S7G, H, J), the NeuN^+^ population actually decreased in both genotypes (Supplementary Fig. S7G–I). Additionally, consistent with the decrease in neurons from P0 (increased neurons in *Pten*^+/−^[23]) to P7 (no difference in neuron number) in vivo, primary culture shows a trend to increased NeuN^+^ cells in *Pten*^+/−^ cultures at DIV2 that has been eliminated by DIV6 (Supplementary Fig. S7G–I).

### Pharmacological rescue of increased glia proliferation in primary cultures derived from *Pten* haploinsufficient mice

To assess proliferation in these cultures, we used 24-h BrdU pulse-chase assays, in which cells that proliferate during the 24-h period express BrdU. We found that DIV8 cultures derived from individual *Pten*^+/−^ mice showed a higher percentage of BrdU^+^ cells that were also Ki67^+^ (BrdU^+^Ki67^+^/BrdU^+^; Ki67 is a marker of cells active in the cell cycle; Fig. 4k, l). Interestingly, 24 h treatment with PI3K inhibitor LY294002 increased BrdU^+^Ki67^+^/BrdU^+^ ratios in *Pten*^+/+^, but not *Pten*^+/−^ cultures, and genotype differences observed in vehicle-treated cultures also remained significant (Fig. 4k, l). It is possible, though, that the increase in *Pten*^+/+^ cultures was due to cell death caused by 10 μM of LY294002: if cells that had undergone division but not re-entered the cell cycle were dying, the number of BrdU^+^ cells would decrease, thus exaggerating the percent of cells re-entering the cell cycle. This possibility will require further examination using apoptotic markers with titrated LY294002 treatment.

Following 24 h treatment with PTEN-Long (a translational variant of PTEN), BrdU^+^Ki67^+^/BrdU^+^ cells were reduced in *Pten*^+/−^ cultures to *Pten*^+/+^ levels, while *Pten*^+/+^ cultures were unaffected (Fig. 4k, l). To confirm that this effect was due to PTEN-Long, we first demonstrated that it is capable of entering the cell (Supplementary Fig. S8A). An antibody blocking test in *Pten*^+/−^ cultures showed a reduction in the effect of PTEN-Long treatment (Supplementary Fig. S8B), although not to vehicle levels. This could be due to the antibody molar concentration, which was selected based on the 75 kDa PTEN-Long protein alone, without accounting for degradation products in our protein preparation (as seen in western blot using the same antibody; Supplementary Fig. S8C).

As we see increased cell cycle re-entry (increased BrdU^+^Ki67^+^/BrdU^+^), we sought to determine if this increased proliferation was present in glial cells. Thus, we stained cultures from *Pten*^+/+^ and *Pten*^+/−^ cortices with antibodies for BrdU and either Olig2 or Sox9. We found that *Pten*^+/−^ cultures showed higher percentages of Olig2^+^ and Sox9^+^ cells that were colabeled with BrdU (Olig2^+^BrdU^+^/Olig2^+^ and Sox9^+^BrdU^+^/Sox9^+^, respectively) at DIV12 (Fig. 4m–p). This effect was suppressed in both Olig2^+^ and Sox9^+^ cells by 24 h LY294002 treatment, which also reduced Sox9^+^BrdU^+^/Sox9^+^ to below untreated *Pten*^+/+^ levels in both genotypes (Fig. 4m–p). Following 24 h PTEN-Long treatment, the *Pten*^+/−^ increase was eliminated in Olig2^+^BrdU^+^/Olig2^+^ (Fig. 4m, n), but not in Sox9^+^BrdU^+^/Sox9^+^, as levels were reduced in both genotypes (Fig. 4o, p).

These results demonstrate that primary cortical cultures derived from *Pten*^+/−^ mice have increased cell cycle re-entry and proliferation of Olig2^+^ and Sox9^+^ cells compared to *Pten*^+/+^ mice, and confirmed that this is through PI3K activity. We also show for the first time that 24 h exposure to exogenous PTEN-Long is sufficient to partially rescue the hyperproliferation phenotype caused by *Pten* haploinsufficiency.

## Discussion

**Fig. 5:**
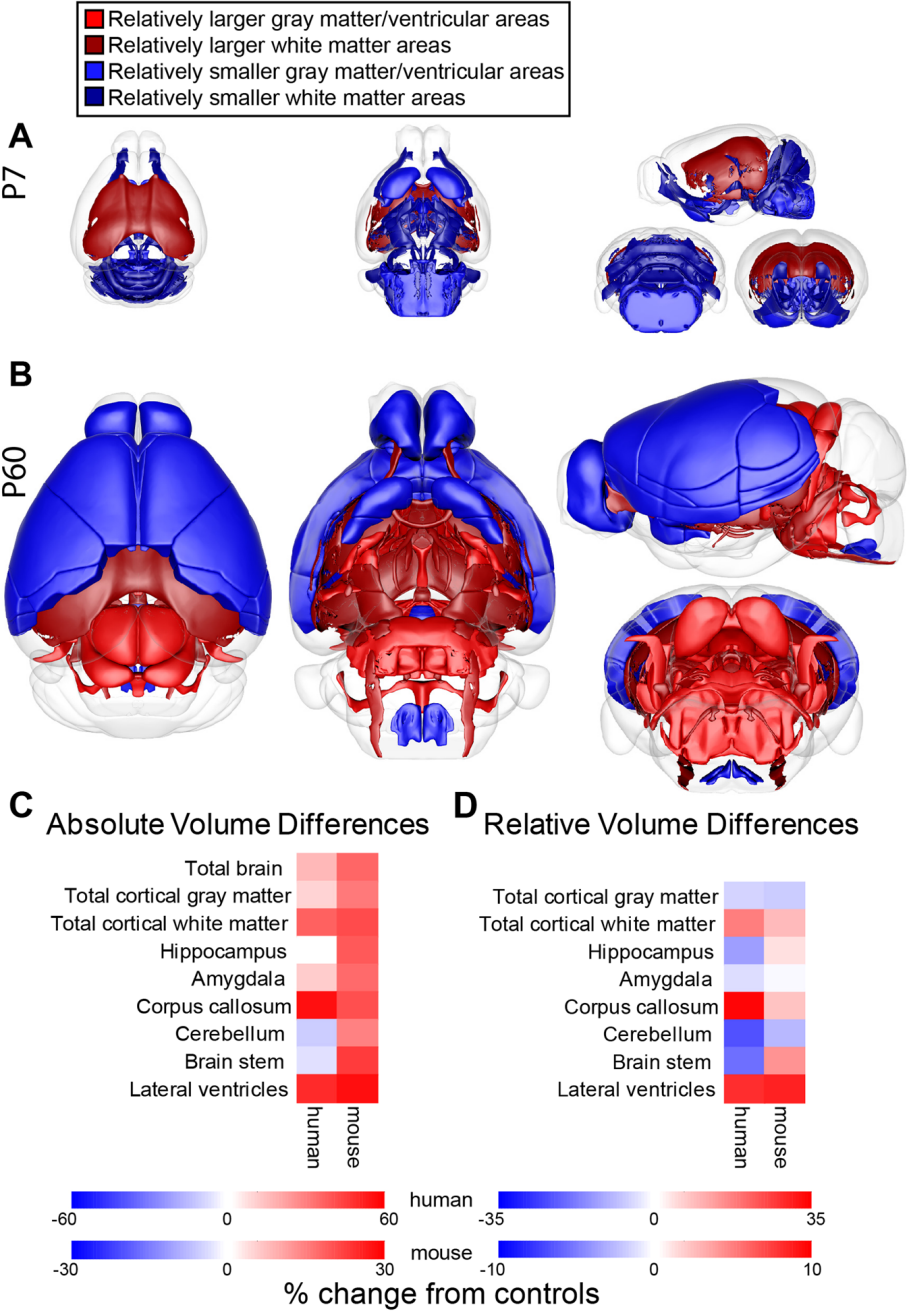
*Pten*^+/−^ mice show similar patterns of overgrowth to humans with autism spectrum disorder and *PTEN* mutations (*PTEN-*ASD). **a**, **b** Overview of relative volume differences in P7 (**a**) and P60 (**b**) mice. Red, relatively larger gray matter and ventricular areas; dark red, relatively larger white matter areas; blue, relatively smaller gray matter and ventricular areas; dark blue, relatively smaller white matter areas. **c**, **d** Comparison of absolute (**c**) and relative (**d**) volume differences from controls in *Pten*^+/−^ mice and *PTEN-*ASD humans. Human data is from Frazier et al.^14^. For mice, total cortical gray matter includes: entorhinal cortex and frontal, occipital, and parieto-temporal lobes; hippocampus includes: dentate gyrus of hippocampus, stratum granulosum of hippocampus, pre-para subiculum, and hippocampus; brain stem includes: medulla, pons, pontine nucleus, and inferior and superior olivary complexes; and cerebellum is cerebellar cortex. Color intensity indicates the magnitude of the percent increase (red) or decrease (blue) from controls.

Germline *Pten* haploinsufficiency results in increased absolute brain volume at both P7 and P60. Our detailed MRI analysis revealed that while absolute volume increases are virtually ubiquitous, they are not uniform across all brain regions; some areas show abnormal scaling (i.e., a change in the percent of the brain occupied by a given region; see Fig. 5a, b). In P60 mice, there are genotype differences in relative GM and WM, with relative increases in WM corresponding with relative decreases in GM. The WM increases may be due to increased proliferation of glia in *Pten*^+/−^ mice. Interestingly, decreased GM regions were all located in the forebrain, while the increased regions were weighted towards the mid- and hindbrain, suggesting an anterior/posterior gradient (Fig. 5b, Supplementary Fig. S4A). At P7, however, there are no differences in relative GM or WM volumes, suggesting potential therapeutic opportunities in the early postnatal stage.

### Similarities and differences between patterns of brain overgrowth in germline *Pten*^+/−^ mice and humans with *PTEN* mutations or idiopathic ASD

For *Pten*^+/−^ mice to be a useful model of macrocephaly/autism syndrome and provide insight into neurodevelopmental effects of *PTEN* haploinsufficiency, these effects must be recapitulated across species. Our results suggest that this is the case, as MRI revealed similar patterns of brain overgrowth in *Pten*^+/−^ mice and humans with mutations in *PTEN*^13,15–17^, with one cohort of *PTEN*-ASD humans^14^ showing striking parallels in both absolute and relative volume changes (Fig. 5c, d). Both *Pten*^+/−^ mice and *PTEN*-ASD humans show increased absolute and relative volume of the brain, CC, lateral ventricles, and cortical WM, and increased absolute, but decreased relative, volume of the amygdala, cerebellum, and cortical GM (Fig. 5c, d, ^14^). T2 weighted WM hypointensity, which suggests increased myelination, was also increased in the human study^14^, consistent with our findings in *Pten*^+/−^ mice. Thus, germline *Pten*^+/−^ mice model multiple neuroanatomical phenotypes that are characteristic of *PTEN-ASD*.

While we are studying one specific risk factor, which is specifically relevant to one clinical population, it is also worth considering potential implications of our findings in the broader context of idiopathic ASD. The data presented here are consistent with several aspects of regional volume changes in some cases of idiopathic ASD with increased brain volume (e.g., absolute increases in the cerebral cortex, limbic structures, brainstem, cerebellum; relative decreases in the cerebral cortex)^38–42^, but not others^39,40,42,43^.

A major limitation of existing data on MRI in individuals with ASD and/or *PTEN* mutations is that they typically occur at or after 2 years of age, when ASD is first diagnosed. By using a mouse model of a rare ASD variant, we were able to assess absolute and relative brain volume changes at P7, which is roughly equivalent to human birth^24,25^. Thus, our results can be used to generate hypotheses about neonatal brain structure abnormalities of individuals with *PTEN* mutations (e.g., normally scaled GM and WM, increased idiosyncrasy in brain region volume). These hypotheses could then be tested in high-risk siblings of children with ASD, and potentially lead to early identification and treatment.

It remains unclear how relative volume changes in specific brain regions relate to abnormal behaviors in *Pten* mutant mice and humans with ASD. In individuals with ASD, both idiopathic and with *PTEN* mutations, WM has been correlated with IQ, processing speed, memory deficits, and repetitive behavior^14,42,44^. While we were unable to perform behavioral analyses on the mice used for the MRI analysis, our detailed mapping of neuroanatomical alterations lay the groundwork for using the *Pten*^+/−^ mouse model to examine what abnormal scaling in specific regions means for behavior and cognition. It is also unclear whether observed hardwired abnormalities in structure, and possibly connectivity (e.g., ^45^) cause irrevocable deficits, or if manipulating plasticity and/or firing rate, for example, can rescue these impairments. Unraveling this question is crucial as we search for a cure or prevention strategy.

### *Pten* mutations result in increased glial proliferation

The WM overgrowth we observed is consistent with our previous results, that glia (including oligodendrocytes), but not neurons, are over-represented in adult *Pten*^+/−^ mice^23^. However, we previously observed the opposite phenotype at P0, with hyperplasia in neuronal but not glial populations^23^. Here we show that the mature pattern has already been established in *Pten*^+/−^ mice by P7, narrowing the window for the neuronal-to-glial hyperplasia switch. Pten has been implicated in the modulation of astrocytes^23,46–48^, microglia^23,49^, and oligodendrocytes^14,23,47,50^; our current data suggest that Pten is acting during the first week of postnatal life to decrease the number of neurons via apoptosis (which is elevated in *Pten*^+/−^ mice at P4^23^) and to increase glial number via increased cell cycle re-entry and proliferation. Consistently, both germline *Pten*^+/−^ and *Pten*^*m3m4/m3m4*^ mice (which have a reduction in the ratio of nuclear to cytoplasmic Pten localization), show increased numbers of putative astrocytes (S100β^+^), cells of oligodendrocyte lineage (Olig2^+^), and CC thickness at or near adulthood^23,47^. *Pten*^*m3m4/m3m4*^ mice also show increased glial fibrillary active protein (GFAP) signal intensity, proliferation of polydendrocyte glia, and upregulation of myelination genes^14,47^. The contributions of pathways downstream of Pten to this phenotype are as yet undetermined. Deletion of the mTOR regulator *Tsc* in neurons led to the opposite result, with decreased myelin binding protein, oligodendrocyte maturation, and myelination in the brain^51^. These differences may be due to gene dosage effects, or to Pten actions on other downstream pathways (e.g., β-catenin, ERK).

In addition to confirming increased glial proliferation in *Pten*^+/−^ mice, our cell culture experiments provide the first evidence that exogenous administration of recombinant PTEN-Long is sufficient to decrease cell cycle re-entry in *Pten*^+/−^ cultures to *Pten*^+/+^ levels without showing signs of cell death, and to suppress proliferation of both Olig2^+^ and Sox9^+^ cells. These data illustrate the potential of PTEN-Long as protein replacement therapy for individuals with *PTEN* mutations, although dosages and bioavailability of PTEN-Long must be tested in preclinical in vivo models before it can be considered a viable therapeutic strategy.

### *Pten* mutations desynchronize growth across areas during development

We found both age- and genotype-related differences in the consistency of brain region volume. Adults could be clearly segregated into genotypes by unbiased clustering of individual mouse deviations from the age-group mean for each brain region, while this method could not similarly segregate P7 mice. This is consistent with the lack of relative changes in overall WM or GM, and our previous finding that P7 *Pten*^+/−^ mice show the smallest postnatal difference in brain size from littermate controls^23^. This is likely due to increased variation across brain regions, which is significantly higher in P7 than P60 mice for both genotypes, with a further increase in P7 *Pten*^+/−^ mice. These data suggest that *Pten* mutations may desynchronize growth across brain regions and animals during early developmental stages, but become more homogenous over time, resulting in stable and consistent structural differences by adulthood. While the mechanism of increased variation is unknown, it could be due to interactions between *Pten* haploinsufficiency and epigenetic and/or environmental factors during early development.

Increased variability in *Pten*^+/−^ mice at this early developmental timepoint could lay the groundwork for later idiosyncratic connectivity. This idiosyncrasy has been observed in resting-state functional MRI in adults with ASD, who show more idiosyncratic patterns of connectivity, activation patterns in response to social videos, and positive correlations between ASD symptoms and “distortion” of functional connectivity (in comparison to canonical pattern in controls)^52–57^. While we have previously shown abnormal prefrontal cortex to basolateral amygdala connectivity in *Pten*^+/−^ adult mice^45^, further examination of the idiosyncrasy of functional connectivity in *Pten*^+/−^ mice during early postnatal development and adulthood is necessary to determine the association between developmental structural idiosyncrasy and the development of functional connectivity.

## Conclusions

Here we show that *Pten*^+/−^ mice show similar patterns of regional brain overgrowth to humans with *PTEN*-ASD, particularly in terms of relative WM enlargement in adulthood. Increased proliferation of glia via reduced suppression of the PI3K pathway likely contributes to this phenotype. Early postnatal *Pten*^+/−^ mice showed no differences in relative WM or GM volume, and increased variability among mice and brain regions in comparison to both age-matched controls and *Pten*^+/−^ adults. These data suggest that brain region growth may be desynchronized across animals and individual brain regions during early developmental stages before stabilizing into more consistent, homogenous neuroanatomical differences by adulthood.

## Supplementary information

Supplemental Materials

Figure S1

Figure S2

Figure S3

Figure S4

Figure S5

Figure S6

Figure S7

Figure S8

Table S1

Table S2
