# Supplementary material for: Pten haploinsufficiency disrupts scaling across brain areas during development in mice
Source: Transl Psychiatry. 2019 Dec 5;9:329. doi: 10.1038/s41398-019-0656-6 (PMC6895202; doi:10.1038/s41398-019-0656-6)
Supplement: Supplementary file 1 — Supplemental Materials [file 41398_2019_656_MOESM1_ESM.docx]

**Supplementary Methods**

**Subjects**

*B6.129-Pten^tm1Rps^* mice^1^ were obtained from the National Cancer Institute repository at Frederick, where they had been backcrossed onto a congenic C57BL/6J background by the Donating Investigator. The line has been maintained by backcrossing to C57BL/6J mice for more than 10 generations. Mice used in this study were males generated by mating *Pten^tm1Rps/+^* (*Pten*^+/-^) male mice with wild-type (*Pten*^+/+^) females. After weaning, mice were housed as previously described^2^ in single-sex groups of 3-5 mice per cage.

**Magnetic Resonance Imaging**

**Brain Collection.** Mice were anesthetized (postnatal day 7, P7: wet ice; P60: avertin [250mg/kg tribromoethanol, VWR, West Chester, PA]), then intracardially perfused with 1X PBS (P7: 4ml; P60: 30ml; Life Technologies, Carlsbad, CA) containing 10U/ml heparin (Sigma-Aldrich, St. Louis, MO) and 2mM ProHance (a Gadolinium contrast agent; Bracco Diagnostics, Monroe Twp, NJ), followed by 4% paraformaldehyde (PFA, Sigma-Aldrich) containing 2mM ProHance (P7: 7ml; P60: 30ml)^3, 4^. After perfusion, mice were decapitated, and skin, lower jaw, ears, and cartilaginous nose tip were removed. The brain and remaining skull structures were incubated in 4% PFA + 2mM ProHance overnight at 4°C then transferred to 1X PBS containing 2mM ProHance and 0.02% sodium azide (Sigma-Aldrich) for ~1 month prior to magnetic resonance imaging (MRI) scanning.

**Imaging.** A multi-channel 7.0 Tesla MRI scanner imaged brains within skulls. Sixteen custom-built solenoid coils allowed imaging of 16 brains in parallel^5^. Parameters for anatomical MRI scans were as follows: T2-weighted, 3-D fast spin-echo sequence, with cylindrical acquisition of k-space, and with a TR of 350ms, and TEs of 12ms per echo for 6 echoes, field-of-view of 20x20x25mm^3^ and matrix size of 504x504x630mm^3^, giving an image with 0.040mm isotropic voxels. Total imaging time is currently 14h^6^.

**Registration and Analysis.** To visualize and compare any changes in mouse brains, images were linearly (6 parameter followed by a 12 parameter) and non-linearly registered towards a pre-existing atlas^7^. To create a population atlas representing the average anatomy of the study sample, all scans were resampled with the appropriate transformation and averaged, resulting in all scans being unbiasedly deformed into alignment. To model how deformation fields relate to genotype, the deformations needed to take each individual mouse’s anatomy into this final atlas space were analyzed^8-10^. The Jacobian determinants of the deformation fields were then calculated as measures of volume at each voxel, and significant volume changes were calculated by warping a pre-existing classified MRI atlas onto the population atlas, which allows for the volume of segmented structures encompassing cortical lobes, large white matter (WM) structures (e.g., corpus callosum, CC), ventricles, cerebellum, brain stem, and olfactory bulbs^7^ to be assessed in all brains. Further, measurements can be examined on a voxel-wise basis in order to localize differences within regions or across the brain. Relative volume was calculated as [(brain region volume)/(whole brain volume) x 100], and percent difference between genotypes was calculated as [(average *Pten*^+/-^ volume)-(average *Pten*^+/+^ volume)/(average *Pten*^+/+^ volume) x 100].

**Additional Analyses.** Additional analyses were performed using R packages *dplyr*, *reshape2*, *magrittr*, *ggplot2*, *RColorBrewer*, and *pheatmap* to generate heatmaps and for unbiased clustering analyses. These analyses were performed on the deviation of each mouse from the mean of all mice of the same age OR genotype (either all P7 and all P60, regardless of genotype, or all *Pten*^+/+^ and all *Pten*^+/-^, regardless of age), calculated as z-scores [e.g., z=(volume_mouse_-mean_all P60 mice_)/(standard deviation_all P60 mice_)], and/or coefficients of variation (CV) for each brain region within age+genotype groups [e.g., CV=(SD_P7_ *_Pten+/-_*)/(mean_P7_ *_Pten_*_+/-_)].

**Eriochrome Cyanine R. Staining for Myelin**

Adult male *Pten*^+/+^ (*n*=3) and *Pten*^+/-^ (*n*=4) littermates were anesthetized with avertin and perfused with PBS followed by 4% PFA in PBS for fixation. Brains were kept in PFA for 3 days, rinsed in PBS, and serial 60μm thick sections were obtained with a vibratome (HM 650V, Thermo Fisher Scientific, Asheville, NC). Every consecutive section was mounted on Superfrost/Plus charged slides (VWR) and dried overnight at room temperature. Sections were rehydrated through gradient ethanol into water, and then stained in eriochrome cyanine R. dye solution [0.21 M ferric chloride (Sigma-Aldrich), 0.2% eriochrome cyanine R. (VWR), 0.5% sulphuric acid (Sigma-Aldrich)] for 5-10min until sections were evenly stained^11^. After rinsing sections in water to eliminate remaining unbound dye, they were immersed in 0.05M aqueous borax (Ward’s Science, Rochester, NY) for differentiating myelin for 1-2min, until only pale blue staining of myelin remained. When differentiation was complete, slides were washed in water. Staining was used to identify myelinated areas; the intensity of stain was not used to interpret the extent of myelination, thus avoiding subjective parameters that could be difficult to avoid in the preparation. Sections were imaged on an Olympus VS120 Virtual Slide Microscope (Olympus, Centerville, PA) with a 10x objective in Bright Field mode. Acquired images were quantified for CC width, anterior commissure (AC) width, and fornix area through anterior/posterior axis positions (estimated with The Mouse Brain in Stereotaxic Coordinates, 3^rd^ edition^12^) using Olympus VS-DESKTOP software.

**Isotropic Fractionator and Flow Cytometry**

**Preparation*.*** Briefly, P7 *Pten*^+/+^ (*n*=3) and *Pten*^+/-^ (*n*=3) littermates were decapitated and their heads were immersed overnight in 4% PFA, followed by brain extraction and a further 5 days’ fixation in 4% PFA. Brains were then dissected into cerebral cortex and remainder, and the cerebral cortex was mechanically dissociated in standard solution (1% Triton X-100 in 40mM sodium citrate; Sigma-Aldrich) with a 7ml glass tissue homogenizer (Kontes Glass Inc., Vineland, NJ).

**Isotropic Fractionator Analysis.** Nuclei in the homogenate were stained with 4’,6-diamidino-2-phenylindole dihydrochloride (DAPI; 1:50 for isotropic fractionator, 1:100 for flow cytometry; D3571, Life Technologies) and primary [anti-NeuN antibody (1:500; 104225, Abcam, Cambridge, MA), anti-Olig2 antibody (1:500; AB9610, Millipore, Billerica, MA)], and secondary [Goat anti-Rabbit 488 (1:2000, A11034, Life Technologies), Goat anti-Mouse 488 (1:2000, A21121, Life Technologies)] antibodies. Total number of nuclei (DAPI+ cells) were counted using a hemocytometer (Thermo Fisher Scientific), taking the average of six 0.1μl nuclei samples per brain.

**Flow Cytometry Analysis.** The numbers of cells positive for neuronal nuclei marker NeuN (NeuN+) and oligodendrocyte transcription factor 2 (Olig2+), which is expressed in oligodendrocytes and their precursors^13, 14^, cells were quantified with flow cytometry using a Gallios flow cytometer analyzer (Beckman Coulter, Brea, CA). DAPI fluorescence was collected with a 450/50 filter (405nm laser), and Alexa 488 with a 550 SP filter (488nm laser). After each solution change, the nuclei were collected by a 5min centrifugation at 375g and resuspended thereafter. Nuclei were stained with DAPI, primary and secondary antibodies, then filtered through a 30µm diameter cell strainer (Thermo Fisher Scientific) before analysis. Background fluorescence, determined using a control with only secondary antibody, was subtracted for each labeling. For quantitative signal analysis, the population of interest was gated, and mean and median intensity of fluorescence of each channel were analyzed using FlowJo software (FlowJo LLC, Ashland, OR).

**Cell Culture and Immunocytochemistry**

**Primary Cortical Culture.** Following dissection, cortices from P0 *Pten*^+/+^ (*n*=3) and *Pten*^+/-^ (*n*=3) mice were placed separately into dissecting media [1X HBSS (Life Technologies), 0.1M HEPES (Affymetrix, Santa Clara, CA), 30mM glucose (Sigma-Aldrich)]. The dissecting media was replaced with culture media [DMEM (Life Technologies), 5% fetal bovine serum (Life Technologies), and 1X penicillin-streptomycin-glutamine (Thermo Fisher Scientific)] containing 1.25% papain (Worthington, Lakewood, NJ), and incubated at 37°C for 15min. After removal of the culture media with papain, 1ml of culture media was added and cells were dissociated. The cell suspension was filtered through a 40μm nylon cell strainer and cells were counted using a hemocytometer. Cells from individual mice were separately plated on coverslips pre-coated with poly-D-lysine (Thermo Fisher Scientific) in 12-well plates at a density of 500 000 cells per well and allowed to grow in a 37°C incubator with 5% CO_2_ levels. Half of the culture media was replaced every 3 days, and cells were fixed at indicated days-in-vitro (DIV) using 4% PFA diluted from a 32% aqueous solution (Electron Microscopy Sciences, Hatfield, PA).

**PTEN-Long.** The JpExress404 *PTEN-Long* construct containing a V5/His tag, deposited by Ramon Parsons^15^, was purchased from Addgene (Cambridge, MA) and transformed into BL21 (DE3) Competent *E. Coli* cells (New England Biolabs, Ipswich, MA) and plated on 100µg/ml carbenicillin LB agar plates. 500ml cultures, grown overnight, were used to inoculate 1L of terrific broth (Sigma-Aldrich), stimulated with 0.2mM IPTG (Sigma-Aldrich), and cultured for 12h at 15°C. Bacteria were lysed by sonication in binding/wash buffer (500mM NaCl, 25mM Tris pH 7.6, 40mM Imidazole) with protease inhibitor (cOmplete Protease Inhibitor Cocktail, Sigma-Aldrich) and then centrifuged at 40 000g for 30min. To the lysate (containing extracted protein), we added 3% milk (BioRad, Hercules, CA) and 3ml of prewashed Ni-NTA beads (Qiagen, Germantown, MD). Following 24h of binding at 4°C, beads were washed 3 times in binding/wash buffer and purified protein was recovered with elution buffer (500mM NaCl, 25mM Tris pH 7.5, 500mM Imidazole). Buffer was replaced with 10% captisol (Cydex Pharmaceuticals, La Jolla, CA) using Amicon Ultra-15 centrifugal filters with 50K MWCO (Millipore) and then concentrated to 200µl. Protein was resolved by SDS-PAGE electrophoresis and quantified by comparison to bovine serum albumin (BSA) standard (Pierce, Carlsbad, CA) using Imperial Protein Stain (Thermo Fisher Scientific).

Western blot analysis was performed to visualize binding of anti-PTEN Mouse monoclonal antibody (1:1000; 9556S, Cell Signaling Technology, Danvers, MA) to PTEN-Long and degradation products. To show that exogenous PTEN-Long can enter cells, we treated primary cortical cultures with vehicle or 100nM of PTEN-Long for 30min, then stained cells with anti-V5-Tag Rabbit primary antibody (1:1000; 13202S, Cell Signaling Technology) and Goat anti-Rabbit 594 secondary antibody (1:1000; A11012, Life Technologies).

To determine if a Pten antibody was able to reduce the effects of PTEN-Long treatment, we incubated vehicle (10% captisol in PBS with 1% BSA) or 1μM PTEN-Long with or without 1μM of PTEN Mouse monoclonal antibody for 1h. The resulting solution was added to primary cortical cultures at 100X for 30min before performing a 24h pulse-chase assay consisting of treating cells with 10μM 5-bromo-2’deoxyuridine (BrdU; B5002, Sigma-Aldrich) for 24h before 4% PFA fixation.

**Culture Treatment and Immunocytochemistry.** To measure cell cycle re-entry and gliogenesis, we performed a 24h BrdU pulse-chase assay by treating cultures at DIV7 or DIV11 with 10μM BrdU and co-applying vehicle (0.1% ethanol; Thermo Fisher Scientific), 10μM phosphoinositide-3-kinase (PI3K) inhibitor LY294002 (S1105, Selleck Chemicals, Houston, TX), or 10nM PTEN-Long. Coverslips from the same animal were randomly assigned to treatments. After 24h treatment, cultures were washed with PBS, fixed with 4% PFA, and stained with primary [anti-BrdU (1:500; ab6326, Abcam), anti-Ki67 (1:1000; ab15580, Abcam), anti-Olig2 (1:1000), anti-Sox9 (1:500; AF3075, R&D Systems, Minneapolis, MN), anti-NeuN (1:1000; MAB377, Millipore)] and secondary [Goat anti-Rat 594 (1:1000; A11007, Life Technologies), Goat anti-Rabbit 488 (1:1000), Goat anti-Mouse (1:1000), Chicken anti-Goat IgG 647 (1:1000; A21469, Life Technologies), Donkey anti-Rat 488 (1:1000; A21208, Life Technologies), Donkey anti-Rabbit 594 (1:1000; A21207, Life Technologies)] antibodies. Images were acquired with the Olympus VS120 microscope and quantified using Olympus VS-DESKTOP software.

For anti-BrdU staining, DNA hydrolysis was performed by incubating cells in 2M HCl for 45min at 37°C, followed by neutralization with 0.1M sodium borate buffer (pH 8.5) for 30min. Cultures were mounted on slides using Vectashield HardSet (Vector Laboratories, Burlingame, CA).

Cell cycle re-entry was quantified at DIV8 by calculating the ratio of BrdU^+^, Ki67^+^ cells to total number of BrdU^+^ cells (BrdU^+^, Ki67^+^/BrdU^+^). Glia proliferation was analyzed at DIV12 by calculating the percentage of BrdU^+^ cells expressing either Olig2 (Olig2^+^BrdU^+^/BrdU^+^) or Sex determining region Y-box 9 (Sox9; a transcription factor that can serve as a nuclear marker for forebrain astrocytes outside neurogenic regions^16, 17^; Sox9^+^BrdU^+^/BrdU^+^). The percentage of all DAPI cells expressing Olig2 or Sox9, as well as the density of cells positive for these markers, were also measured. To confirm that neurons are not proliferating in culture under the conditions used, we calculated the density and percentage of NeuN^+^ cells at DIV2 and DIV6.

**Statistical Analysis**

For all analyses of variance (ANOVAs), Tukey’s or Sidak corrected *post hoc* tests were used when warranted. The α level was set at 0.05, all tests were two-tailed, and all statistics were performed after passing normality tests using PASW 18 (IBM Corporation, Armonk, NY). Results are presented in Supplementary Table S2, and details of individual analyses are listed below. All measurements were performed blind to genotype and/or treatment.

**Brain Volume, Grey and White Matter Volume, Body Mass, Isotropic Fractionator, Flow Cytometry.** Independent-sample *t*-tests were used to compare genotypes for these data. Litter effects were assessed using one-way ANOVAs for brain volume and body mass at each age independently.

**Additional MRI Analyses.** Two-way ANOVAs were used to compare genotype x age effects on CVs in the MRI data across all areas, as well as across grey matter only and WM only.

**Eriochrome Cyanine R. Staining for Myelin.** For the analysis of WM tract size by myelin staining, we collapsed corpus callosum data into two macro data points, pre- (anterior to) and post- (posterior to) Bregma, since it was not possible to correctly determine equality of covariance matrices (Box’s test) or sphericity (Mauchly’s test), likely due to multiple redundant data points. For the purposes of comparison, we also collapsed anterior commissure data into similar macro data points. Thus, these data were analyzed using two-way mixed model ANOVAs for genotype and anatomical area. Planned comparisons (independent-sample *t*-tests) were used to compare genotypes at the anterior and posterior positions, as well as the average width of the CC and AC, and the area of the fornix.

**Cell Culture: NeuN and DAPI at Multiple DIVs.** Genotype x DIV ANOVAs were used, with planned comparisons to assess differences between genotypes at each DIV (independent-sample *t*-tests) and the rate of change across DIV (paired-sample *t*-tests for NeuN^+^ and NeuN^+^ as a percent of DAPI^+^; one-way ANOVAs for DAPI^+^).

**Cell Culture: Olig2, Sox9, and DAPI.** Independent-samples t-tests were used to compare genotypes for the density and percentage of Olig2^+^ and Sox9^+^ cells.

**Cell Culture: BrdU Pulse-Chase Experiments.** Genotype x treatment ANOVAs analyzed the effects of PI3K inhibitor LY294002 and PTEN-Long in *Pten*^+/+^ and *Pten*^+/-^ cultures. Planned comparisons were performed for genotype (independent-samples *t*-tests) and treatment (one-way ANOVAs). An antibody x PTEN-Long ANOVA was also performed, as were planned comparisons for the effects of antibody and treatment (independent-samples t-tests).

**References**

1. Podsypanina K et al. Mutation of *Pten*/*Mmac1* in mice causes neoplasia in multiple organ systems. *Proc Natl Acad Sci U S A* 1999; **96**(4)**:** 1563-1568.

2. Clipperton-Allen AE, Page DT. *Pten* haploinsufficient mice show broad brain overgrowth but selective impairments in autism-relevant behavioral tests. *Hum Mol Genet* 2014; **23**(13)**:** 3490-3505.

3. Spring S, Lerch JP, Henkelman RM. Sexual dimorphism revealed in the structure of the mouse brain using three-dimensional magnetic resonance imaging. *NeuroImage* 2007; **35**(4)**:** 1424-1433.

4. Lerch JP, Sled JG, Henkelman RM. MRI phenotyping of genetically altered mice. *Methods Mol Biol* 2011; **711:** 349-361.

5. Bock NA, Nieman BJ, Bishop JB, Mark Henkelman R. In vivo multiple-mouse MRI at 7 Tesla. *Magn Reson Med* 2005; **54**(5)**:** 1311-1316.

6. Spencer Noakes TL, Henkelman RM, Nieman BJ. Partitioning k-space for cylindrical three-dimensional rapid acquisition with relaxation enhancement imaging in the mouse brain. *NMR Biomed* 2017; **30**(11).

7. Dorr AE, Lerch JP, Spring S, Kabani N, Henkelman RM. High resolution three-dimensional brain atlas using an average magnetic resonance image of 40 adult C57Bl/6J mice. *NeuroImage* 2008; **42**(1)**:** 60-69.

8. Lerch JP et al. Automated cortical thickness measurements from MRI can accurately separate Alzheimer's patients from normal elderly controls. *Neurobiol Aging* 2008; **29**(1)**:** 23-30.

9. Nieman BJ, Flenniken AM, Adamson SL, Henkelman RM, Sled JG. Anatomical phenotyping in the brain and skull of a mutant mouse by magnetic resonance imaging and computed tomography. *Physiol Genomics* 2006; **24**(2)**:** 154-162.

10. Nieman BJ et al. MRI to Assess Neurological Function. *Curr Protoc Mouse Biol* 2018; **8**(2)**:** e44.

11. Kiernan JA. Chromoxane cyanine R. II. Staining of animal tissues by the dye and its iron complexes. *J Microsc* 1984; **134**(Pt 1)**:** 25-39.

12. Franklin KBJ, Paxinos G. *The Mouse Brain in Stereotaxic Coordinates*, 3rd edn. Elsevier: New York, 2008.

13. Shimizu T et al. Olig2-lineage cells preferentially differentiate into oligodendrocytes but their processes degenerate at the chronic demyelinating stage of proteolipid protein-overexpressing mouse. *J Neurosci Res* 2013; **91**(2)**:** 178-186.

14. Zhou Q, Wang S, Anderson DJ. Identification of a novel family of oligodendrocyte lineage-specific basic helix-loop-helix transcription factors. *Neuron* 2000; **25**(2)**:** 331-343.

15. Hopkins BD et al. A secreted PTEN phosphatase that enters cells to alter signaling and survival. *Science* 2013; **341**(6144)**:** 399-402.

16. Sun W et al. SOX9 Is an Astrocyte-Specific Nuclear Marker in the Adult Brain Outside the Neurogenic Regions. *J Neurosci* 2017; **37**(17)**:** 4493-4507.

17. Pompolo S, Harley VR. Localisation of the SRY-related HMG box protein, SOX9, in rodent brain. *Brain Res* 2001; **906**(1-2)**:** 143-148.

**Supplementary Figure Legends**

**Figure S1.** Absolute and relative volumes of additional forebrain grey matter regions in postnatal day 7 (P7; *Pten*^+/+^ *n*=10; *Pten*^+/-^ *n*=10) and P60 (*Pten*^+/+^ *n*=10; *Pten*^+/-^ *n*=9) *Pten*^+/+^ (white bars, white circles) and *Pten*^+/-^ (black bars, black triangles) mice. The parieto-temporal lobe of the cerebral cortex (**A**) showed a trend to decreased relative volume in P60 *Pten*^+/-^ mice. All remaining forebrain grey matter regions showed no relative volume changes (amygdala, **B**; basal forebrain, **C**; bed nucleus of the stria terminalis, **D**; cerebral cortex: entorhinal cortex, **E**; cerebral cortex: occipital lobe, **F**; dentate gyrus of the hippocampus, **G**; hippocampus, **H**; hypothalamus, **I**; lateral septum, **J**; mammillary bodies, **K**; medial septum, **L**; olfactory tubercle, **M**; striatum, **N**; thalamus, **O**). *** *p*<0.001, * *p*<0.05, + *p*<0.1. Mean ± SEM.

**Figure S2.** Absolute and relative volumes of additional brain regions in postnatal day 7 (P7; *Pten*^+/+^ *n*=10; *Pten*^+/-^ *n*=10) and P60 (*Pten*^+/+^ *n*=10; *Pten*^+/-^ *n*=9) *Pten*^+/+^ (white bars, white circles) and *Pten*^+/-^ (black bars, black triangles) mice. **A-E**) Midbrain (**A-B**) and hindbrain (**C-E**) grey matter regions showed increased absolute volume but no relative volume changes (midbrain, **A**; periaqueductal grey, **B**; cerebellar cortex, **C**; pontine nucleus, **D**; superior olivary complex, **E**). The subependymale zone/rhinocele (**F**) was relatively decreased at P7, but all remaining ventricle regions were absolutely increased but relatively unchanged (cerebral aqueduct, **G**; fourth ventricle, **H**). The corticospinal tract/pyramids (**I**) in the lateral forebrain bundle was relatively decreased at P7, and remaining medial forebrain bundles were either relatively decreased (fasciculus retroflexus, **J**; fimbria, **K**) or not relatively changed (anterior commissure, pars posterior, **L**; mammillothalamic tract, **M**). Cranial nerve regions were also relatively decreased at P7 (medial lemniscus/medial longitudinal fasciculus, **N**; lateral olfactory tract, **O**). *** *p*<0.001, ** *p*<0.01, * *p*<0.05, + *p*<0.1. Mean ± SEM.

**Figure S3.** Brain overgrowth was not due to litter effects, as litter did not significantly affect brain mass at postnatal day 7 (P7; *Pten*^+/+^ *n*=10; *Pten*^+/-^ *n*=10; **A**) or P60 (*Pten*^+/+^ *n*=10; *Pten*^+/-^ *n*=9; **B**).There were no genotype differences in body mass between *Pten*^+/+^ (white bars) and *Pten*^+/-^ (black bars) at P7 (**C**) or P60 (**D**), although litter effects on body mass were significant at P7 (**C**), with a statistical trend at P60 (**D**). Different colors denote different litters.

**Figure S4.** Distribution and types of regions with significant differences in relative volume between *Pten*^+/+^ (P7: *n*=10; P60: *n*=10) and *Pten*^+/-^ (P7: *n*=10; P60: *n*=9) vary across developmental stages. **A-B**) Pie charts showing the volume of grey and white matter regions relatively increased (red), relatively decreased (blue), or normally scaled (grey) at postnatal day 60 (P60; **A**) and P7 (**B**), grouped by anatomical region. Slice size represents the volume of the total anatomical region. LFB, lateral forebrain bundle; MFB, medial forebrain bundle; Cranial, cranial nerves; Cerebellar, cerebellar fiber tracts. **C**) Number of brain regions showing significant relative increases (right of 0) and decreases (left of 0) in *Pten*^+/-^ mice at P7 and P60. Grey bars indicate grey matter regions, white bars indicate white matter regions, and pale violet bars indicate ventricles.

**Figure S5.** Unbiased clustering of deviation from genotype group mean shows clear segregation of mice by age in both *Pten*^+/+^ (P7: *n*=10; P60: *n*=10; **A**) and *Pten*^+/-^ (P7: *n*=10; P60: *n*=9; **B**) mice. Deviation is calculated as the z-score from genotype group mean [e.g., z-score = (volume­­­_mouse_ – mean volume­_all_ *_Pten_*_+/+ mice_)/(standard deviation_all_ *_Pten_*_+/+ mice_)]. Top dendrogram indicates unbiased clustering of individual mice (green, postnatal day 7, P7; blue, P60), and left dendrogram indicates unbiased clustering of brain regions.

**Figure S6.** *Pten*^+/-^ mice have more glia, but not more neurons, than *Pten*^+/+^ mice in the cortex at postnatal day 7 (P7). **A-D**) Brain mass (**A**), cortex mass (**B**), and total number of nuclei in cortex (**C**), but not nuclei density in cortex (**D**) were higher in *Pten*^+/-^ (black bars) than *Pten*^+/+^ (white bars) mice. **E**) Olig2^+^ cell number was increased in the cortices of *Pten*^+/-^ over *Pten*^+/+^ mice. All *n*=3. ** *p*<0.01, * *p*<0.05. Mean ± SEM.

**Figure S7.** *Pten*^+/-^ cultures contain a higher density of Olig2^+^ and Sox9^+^ cells at 12 days-in-vitro (DIV12), and the number of NeuN^+^ cells decreases from DIV2 to DIV6. **A-C**) Representative images (**A**) and quantification of the number (**B**) and percentage (**C**) of cells positive for oligodendrocyte marker Olig2 at DIV12. **D-F**) Representative images (**D**) and quantification of the number (**E**) and percentage (**F**) of cells positive for astrocyte marker Sox9 at DIV12. **G-K**) Neurons decrease, but total cells increase, from DIV2 to DIV6. **G-H**) Representative images from DIV2 (**G**) and DIV6 (**H**). **I-K**) Quantification of the density (**I**) and percentage (**K**) of neuronal (NeuN^+^) cells, and total cell density (**J**) in culture. All *n*=3 biological replicates. *** *p*<0.001, ** *p*<0.01, * *p*<0.05, + *p*<0.1. Mean ± SEM. Scale bars, 50μm.

**Figure S8.** PTEN-Long enters the cell and its actions are impaired by Pten antibody blocking. **A**) Anti-V5 staining showing localization of V5-tagged PTEN-Long to the cell body in *Pten*^+/+^ and *Pten*^+/-^ primary cortical cultures following 30 minutes of exogenous treatment with 100nM of PTEN-Long. **B**) Pten antibody decreases the suppression of cell cycle re-entry by PTEN-Long in *Pten*^+/-^ cultures (*n*=3 biological replicates for each). **c**) Western blot (left) of protein preparation showing the mouse anti-Pten antibody binding to the PTEN-Long at 75kDa size and other variants and degradation products. Protein is also visualized with Imperial Protein Stain (right). Ab, antibody. ** *p*<0.01, * *p*<0.05. Mean ± SEM. Scale bars, 50μm.
