# Supplementary figures and images for: Pten haploinsufficiency disrupts scaling across brain areas during development in mice
Source: Transl Psychiatry. 2019 Dec 5;9:329. doi: 10.1038/s41398-019-0656-6 (PMC6895202; doi:10.1038/s41398-019-0656-6)

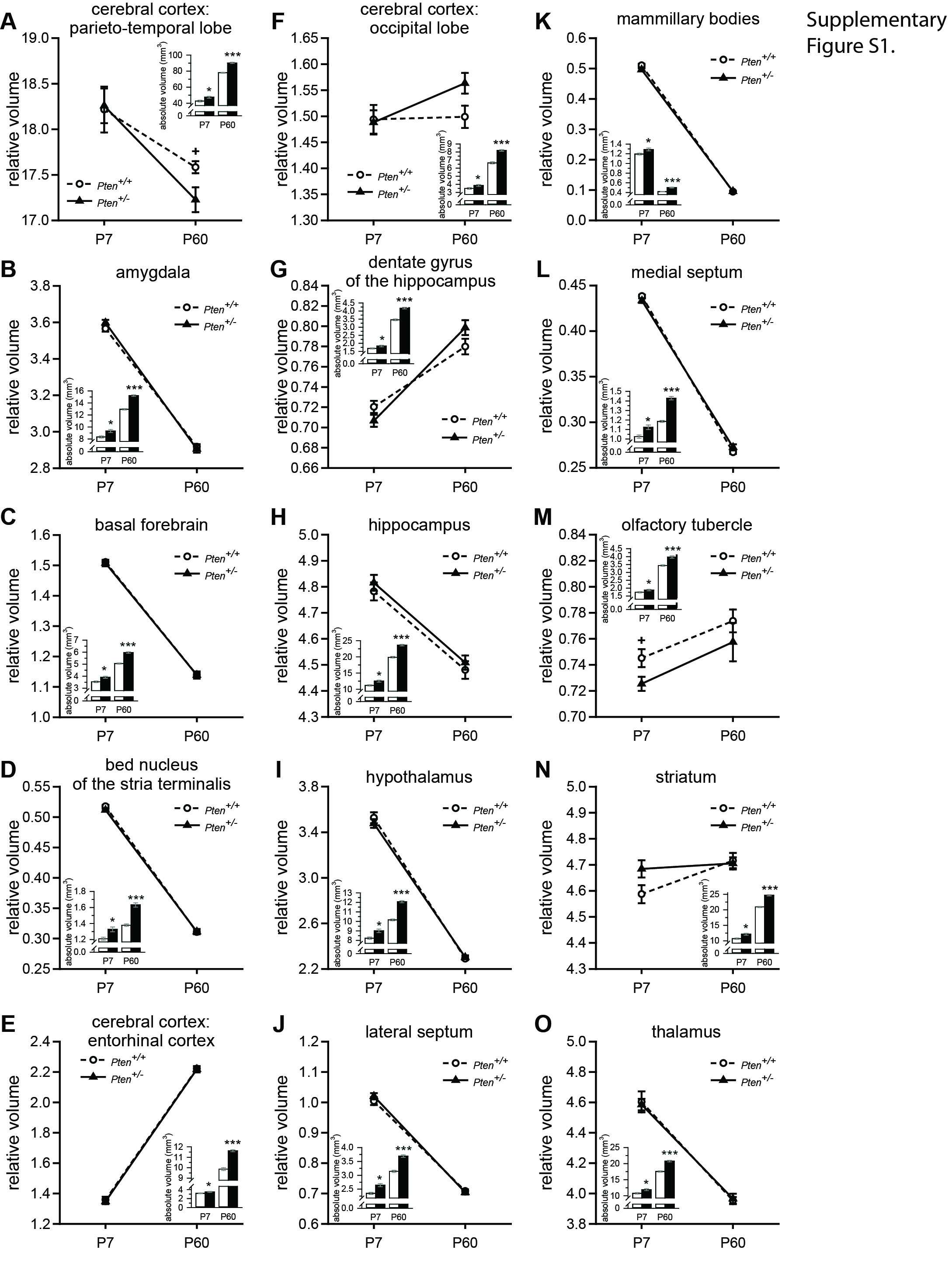

Supplement: Supplementary file 2 — Figure S1 [file 41398_2019_656_MOESM2_ESM.jpg]

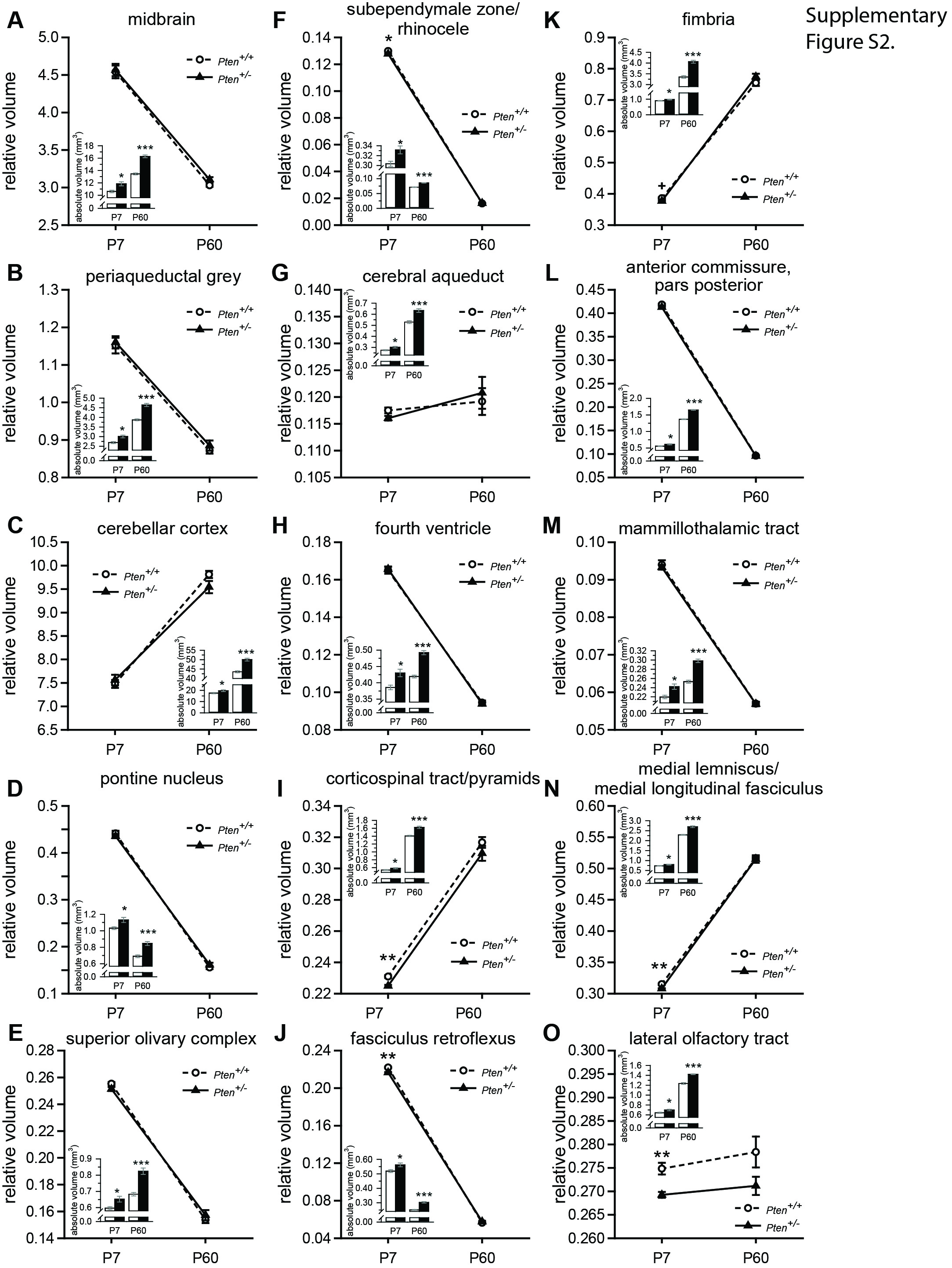

Supplement: Supplementary file 3 — Figure S2 [file 41398_2019_656_MOESM3_ESM.jpg]

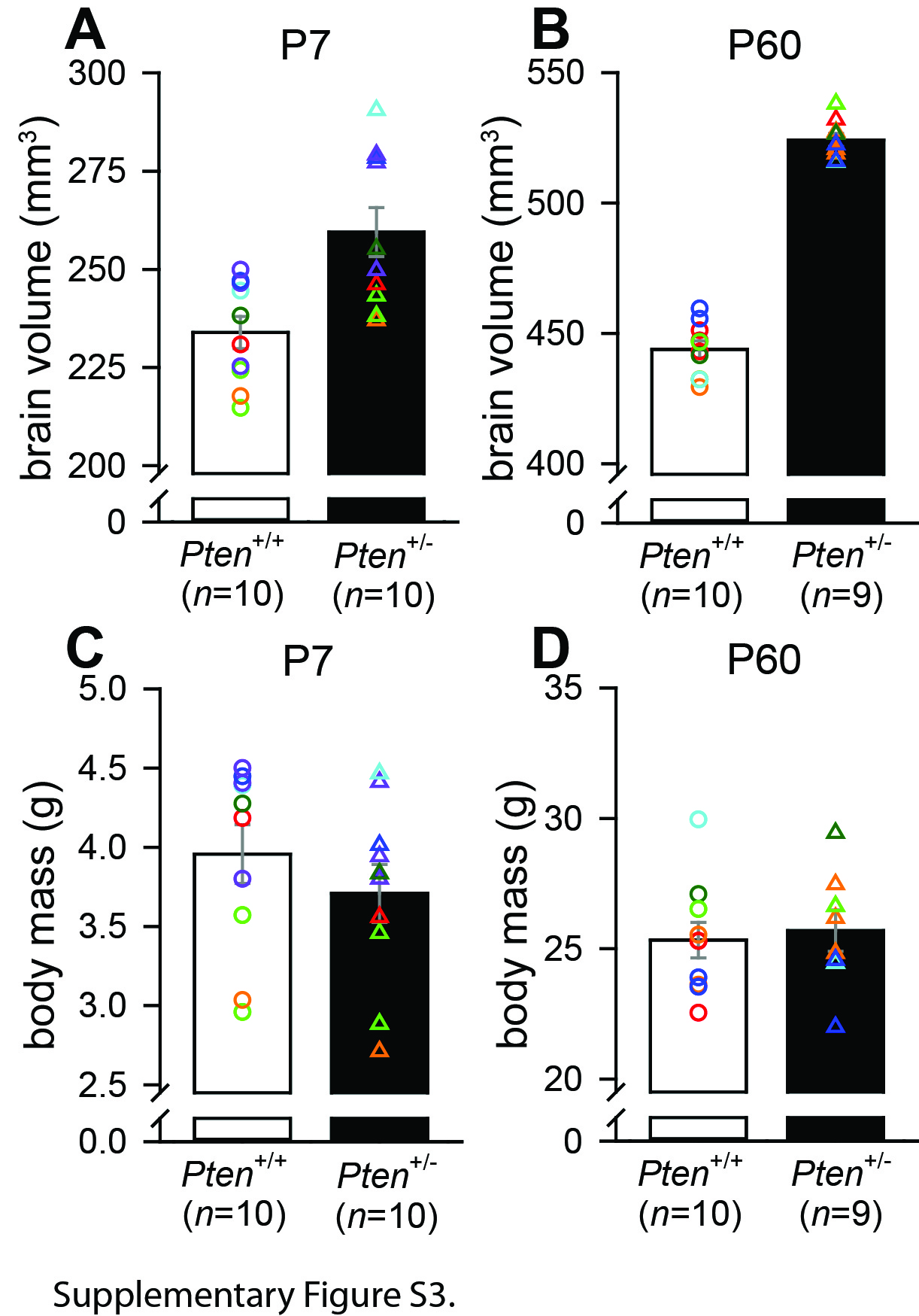

Supplement: Supplementary file 4 — Figure S3 [file 41398_2019_656_MOESM4_ESM.jpg]

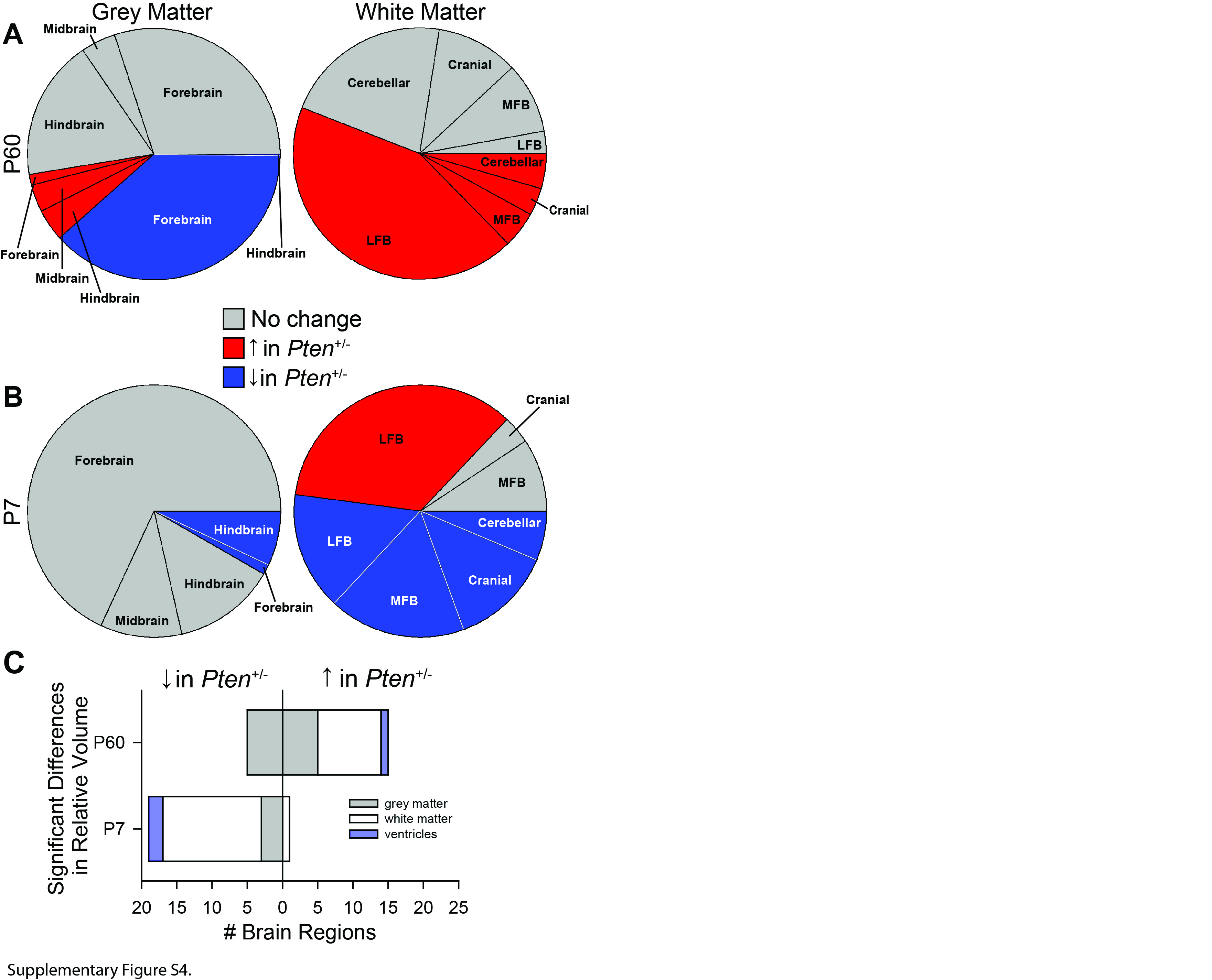

Supplement: Supplementary file 5 — Figure S4 [file 41398_2019_656_MOESM5_ESM.jpg]

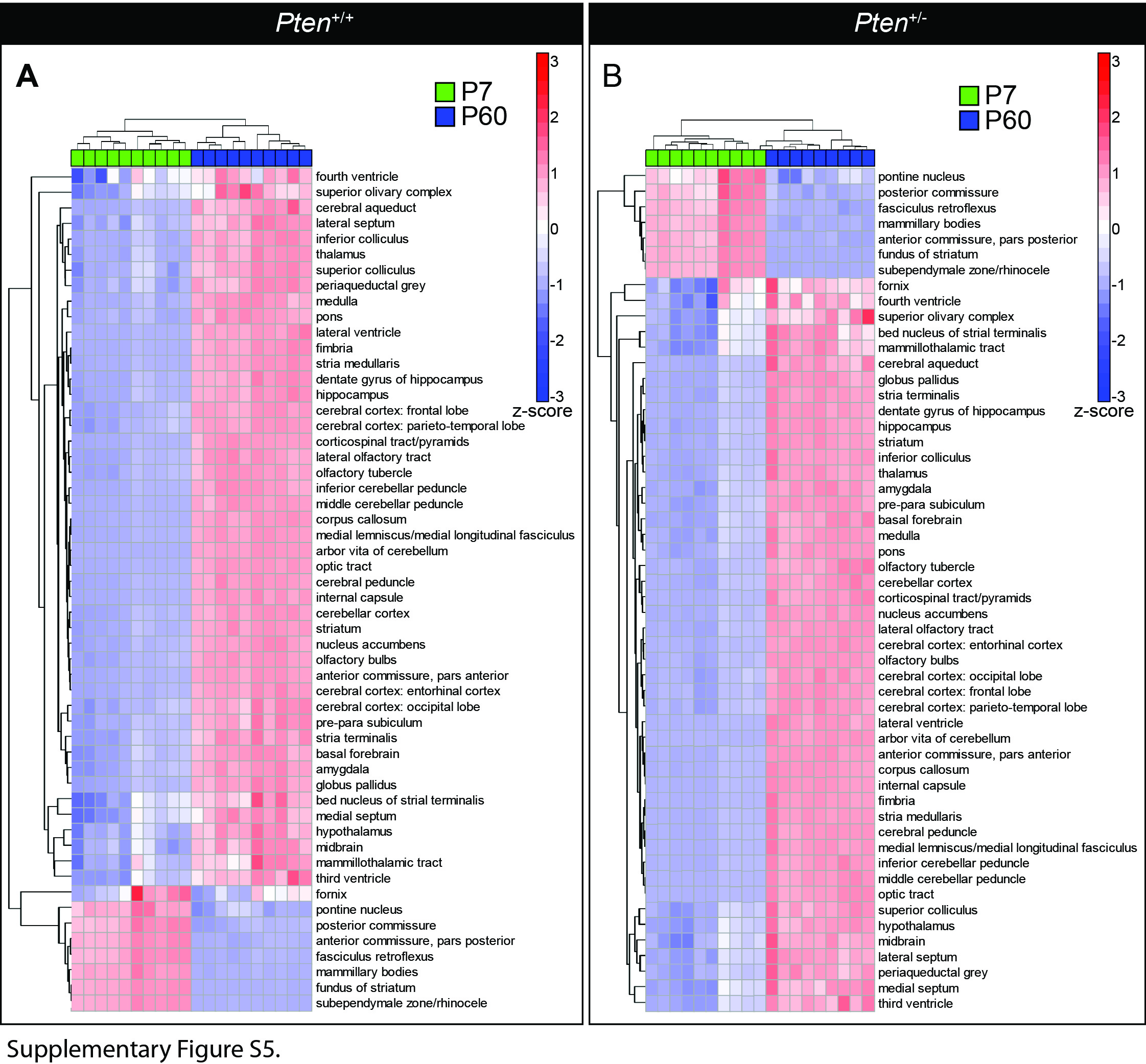

Supplement: Supplementary file 6 — Figure S5 [file 41398_2019_656_MOESM6_ESM.jpg]

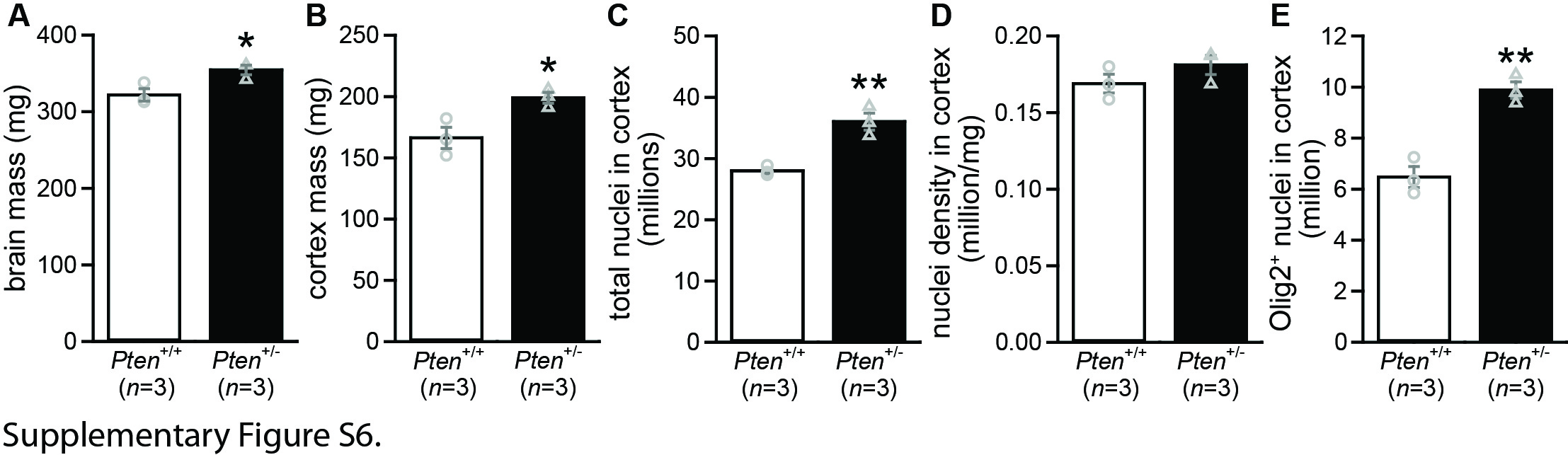

Supplement: Supplementary file 7 — Figure S6 [file 41398_2019_656_MOESM7_ESM.jpg]

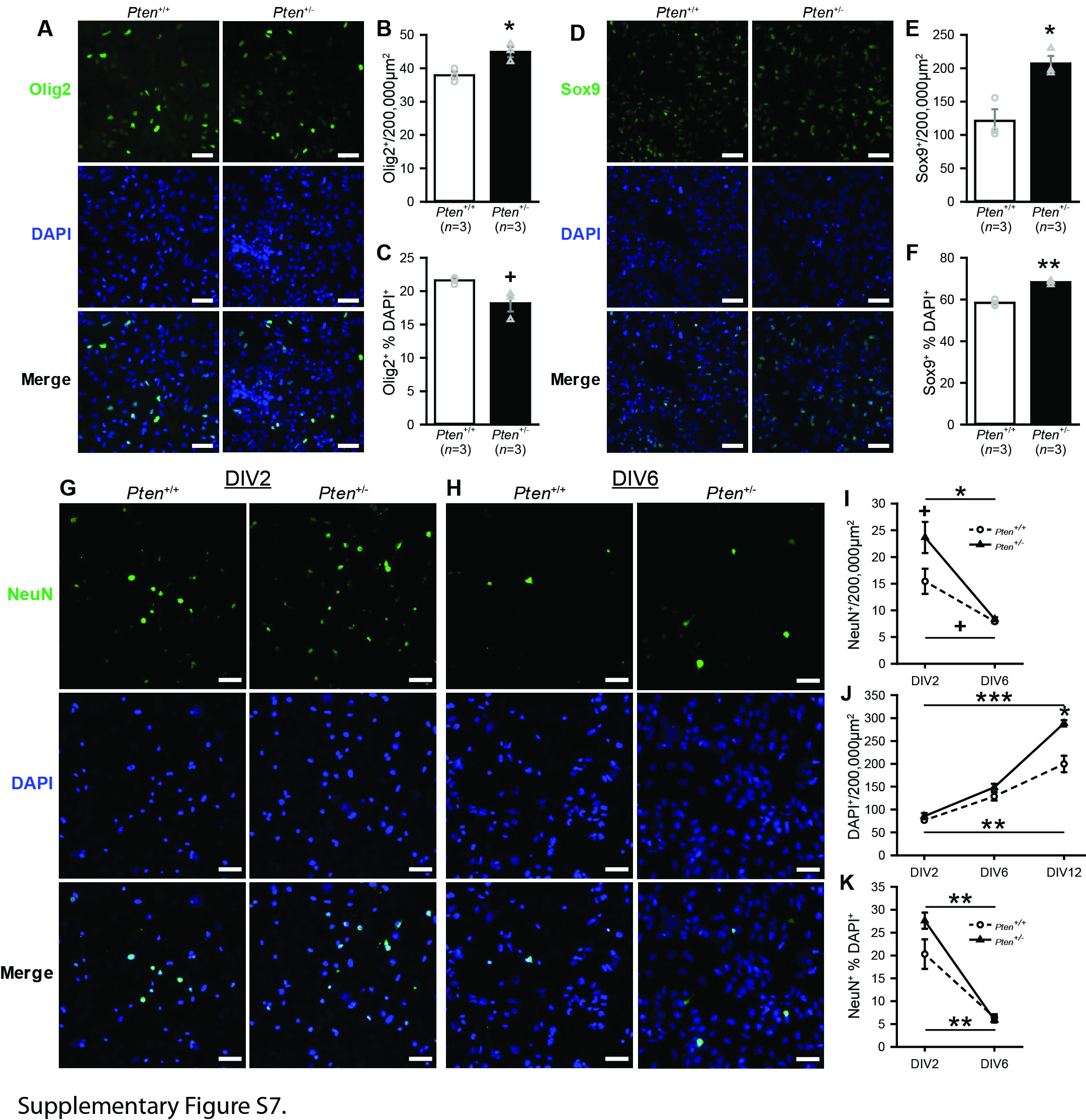

Supplement: Supplementary file 8 — Figure S7 [file 41398_2019_656_MOESM8_ESM.jpg]

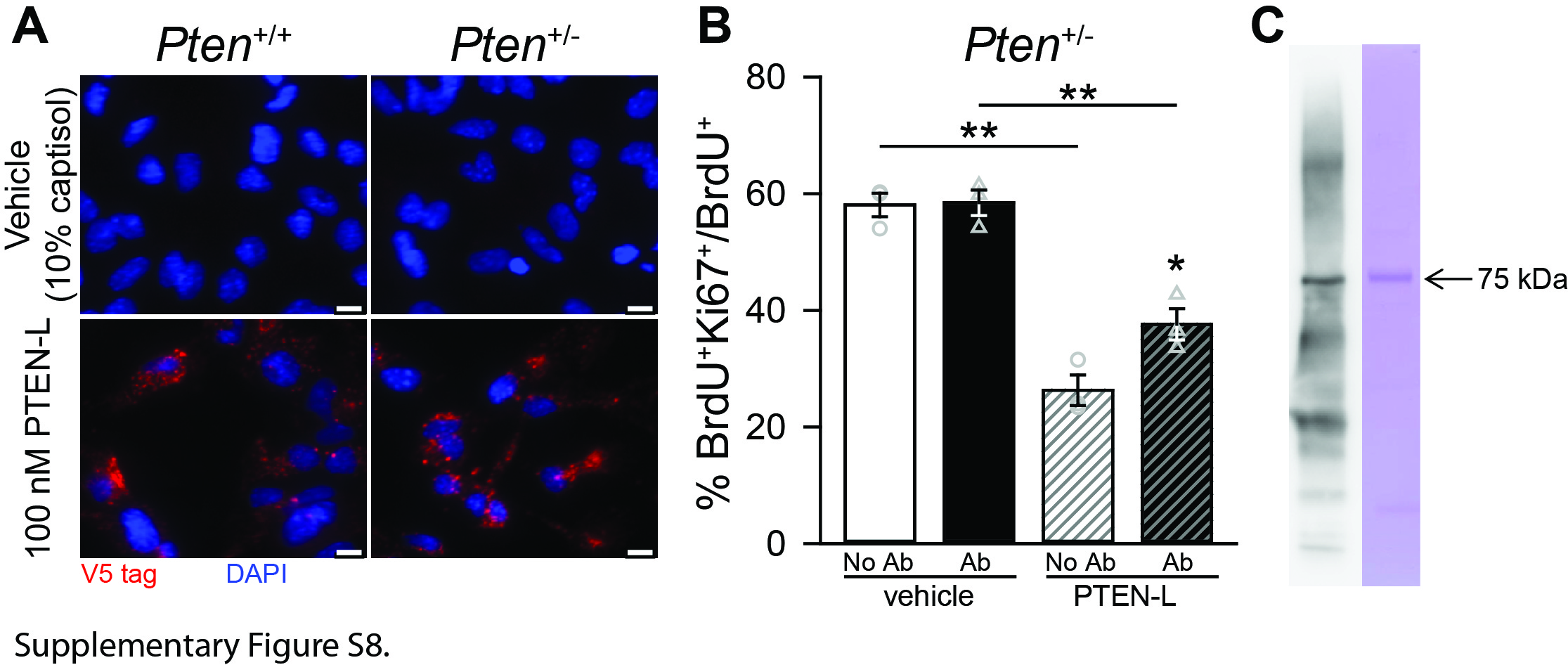

Supplement: Supplementary file 9 — Figure S8 [file 41398_2019_656_MOESM9_ESM.jpg]
